# Nonlinearity-mediated digitization and amplification in electromechanical phonon-cavity systems

**DOI:** 10.1038/s41467-022-29995-x

**Published:** 2022-04-29

**Authors:** Tongqiao Miao, Xin Zhou, Xuezhong Wu, Qingsong Li, Zhanqiang Hou, Xiaoping Hu, Zenghui Wang, Dingbang Xiao

**Affiliations:** 1 College of Intelligence Science, National University of Defense Technology, 410073 Changsha, China; 2 The Laboratory of Science and Technology on Integrated Logistics Support, National University of Defense Technology, 410073 Changsha, China; 3 MEMS Engineering Center of Hunan, 410100 Changsha, China; 4 Institute of Fundamental and Frontier Sciences, University of Electronic Science and Technology of China, 610054 Chengdu, China; 5 State Key Laboratory of Electronic Thin Films and Integrated Devices, University of Electronic Science and Technology of China, 610054 Chengdu, China

**Keywords:** Applied physics, Mechanical engineering

## Abstract

Electromechanical phonon-cavity systems are man-made micro-structures, in which vibrational energy can be coherently transferred between different degrees of freedom. In such devices, the energy transfer direction and coupling strength can be parametrically controlled, offering great opportunities for both fundamental studies and practical applications such as phonon manipulation and sensing. However, to date the investigation of such systems has largely been limited to linear vibrations, while their responses in the nonlinear regime remain yet to be explored. Here, we demonstrate nonlinear operation of electromechanical phonon-cavity systems, and show that the resonant response differs drastically from that in the linear regime. We further demonstrate that by controlling the parametric pump, one can achieve nonlinearity-mediated digitization and amplification in the frequency domain, which can be exploited to build high-performance MEMS sensing devices based on phonon-cavity systems. Our findings offer intriguing opportunities for creating frequency-shift-based sensors and transducers.

## Abstract (web summary)

Electromechanical phonon cavity systems offer advantages for solid state implementation, but thus far investigation has been limited to the linear regime. Here, Miao et al demonstrate non-linear operation of an electromechanical phonon cavity system and the drastic difference to the linear regime.

## Introduction

Nonlinearity is ubiquitous in real-world physical systems. Among the many man-made structures, resonant microelectromechanical and nanoelectromechanical systems (MEMS/NEMS) offer great opportunities for designing, tuning, and exploiting nonlinearities and have enabled the exploration of nonlinear processes and nonlinearity-dictated device properties, such as energy dissipation^1^, energy quantization^2^, superposition of states^3^, dynamic range^4^, and enhancement of frequency stability^5^. As the degree of freedom (DOF) in the system increases (i.e., more than one resonant mode or vibrating part), nonlinearity gives rise to unique phenomena such as synchronization^6–8^, chaos^9^, internal resonance^10^, and generation of frequency comb^11–13^, by enabling and modulating coherent energy transfer between the different DOFs^14^.

Among multi-DOF systems, electromechanical phonon-cavity systems^15^ offer the unique capability of phonon manipulation by parametrically coupling a mechanical resonance to a phonon cavity^16,17^, in many aspects analogous to a photon-cavity^18–22^, which has enabled a plethora of exotic physical phenomena such as self-cooling^23,24^, induced transparency^25,26^, parametric amplification^27^, and quantum squeezing^28,29^. These exquisite functions are realized through a pump signal in the cavity’s sideband, which parametrically controls the dynamical coupling and backaction between the resonant mode and the cavity^30–32^.

One key feature that differentiates the phonon-cavity systems is that they operate entirely in the electromechanical domain, and thus are much more advantageous for implementation using monolithic solid-state devices. Further, the characteristic frequency of the phonon cavity is typically in the same frequency band as the resonant mode (unlike that of a photon-cavity, typically at orders-of-magnitude higher frequency), which greatly simplifies the signal transduction, making such systems promising for frequency-shift-based applications^8,33–35^, such as sensing.

However, in such parametrically-coupled systems, despite the nonlinear nature of the coupling between the different DOFs^14^, research to date has been largely confined to linear operations, i.e., with limited vibration amplitude, which has plagued the exploration of nonlinear processes in these systems. Here we study nonlinear operation in a microelectromechanical phonon-cavity system and show that the response clearly differs from that in the linear regime. More importantly, leveraging the nonlinearity-mediated bi-stability, we demonstrate digitization and amplification modes of signal sensing enhancement in the frequency domain, which can be further tuned by the degree of nonlinearity in the vibration response.

## Results

**Fig. 1:**
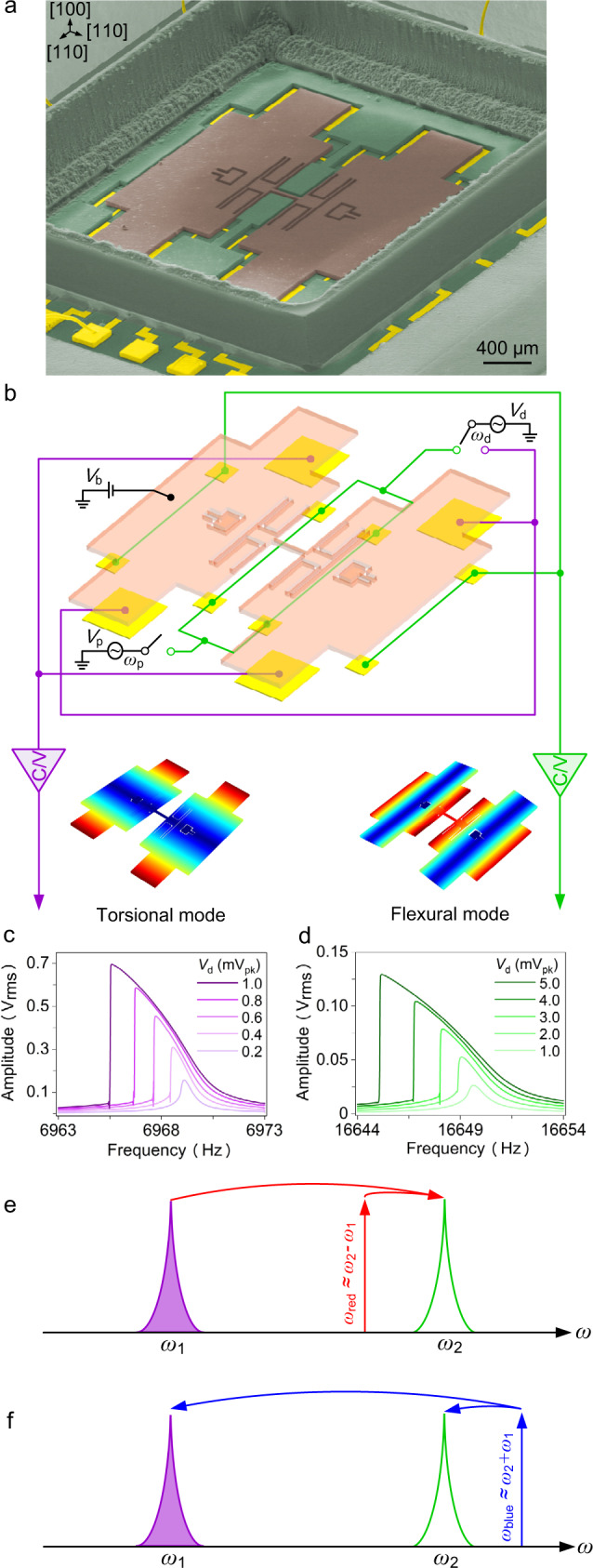
Electromechanical phonon-cavity system with nonlinear responses. **a** A false-color SEM image of the MEMS resonator designed to exhibit two prominent resonant modes. **b** Schematic illustration of the electrode layout underneath the resonator body and the simplified measurement circuit diagram. **c**, **d** Resonant responses (during downward frequency sweeps) of the two modes, showing nonlinear vibration with clear bi-stability (the vertical jump in amplitude), with mode shapes illustrated above. **e** Schematic diagram of the phonon cavity system operating with the parametric pump in the cavity red sideband, and **f** blue sideband.

The electromechanical resonator used in this study (Fig. 1a) is designed to exhibit a torsional mode (at frequency *ω*_1_ = 2*π* × 6969.1 Hz) and a flexural mode resonance (at frequency *ω*_2_ = 2*π* × 16649.6 Hz). In this work, we specifically design our device to facilitate the observation of dynamical coupling in the nonlinear regime. This is achieved by the small capacitance gap, large capacitance area, and high-quality factor of the resonator (see “Methods”). By activating different combinations of the 12 electrodes (Fig. 1b), we can selectively excite these two distinct modes in both linear and nonlinear regimes (Fig. 1c, d), as well as injecting a parametric pump signal that controls the coupling between these two modes (see “Methods” and Fig. S2 for additional details). Enabled by the capacitive transduction scheme, both resonant modes exhibit clear Duffing softening response^36,37^ (see SI 2.2), with clear nonlinearity-mediated bi-stability (vertical jump in amplitude). We denote the critical frequency where such bi-stability occurs as *ω*_b_, which depends on the driving amplitude and the sweep direction (Fig. S3).

The electromechanical structure is designed to facilitate coherent energy transfer between the two resonant modes via parametric pump-controlled intermodal coupling. Among the two resonant modes, the flexural mode at frequency *ω*_2_ serves as the phonon cavity, which can coherently exchange energy with the resonant mode of interest at *ω*_1_ if the pump frequency *ω*_p_ aligns with the red sideband of the cavity at *ω*_red_ = *ω*_2_ − *ω*_1_, or the blue sideband at *ω*_blue_ = *ω*_2_ + *ω*_1_. Specifically, when only the mechanical mode at *ω*_1_ is excited, pumping the red sideband leads to phonon removal at *ω*_1_ (Fig. 1e), while pumping the blue sideband engenders phonon creation (Fig. 1f). This parametrically excited-modulated vibrational energy transfer forms the operation basis of phonon-cavity systems^15,16,38^.

**Fig. 2:**
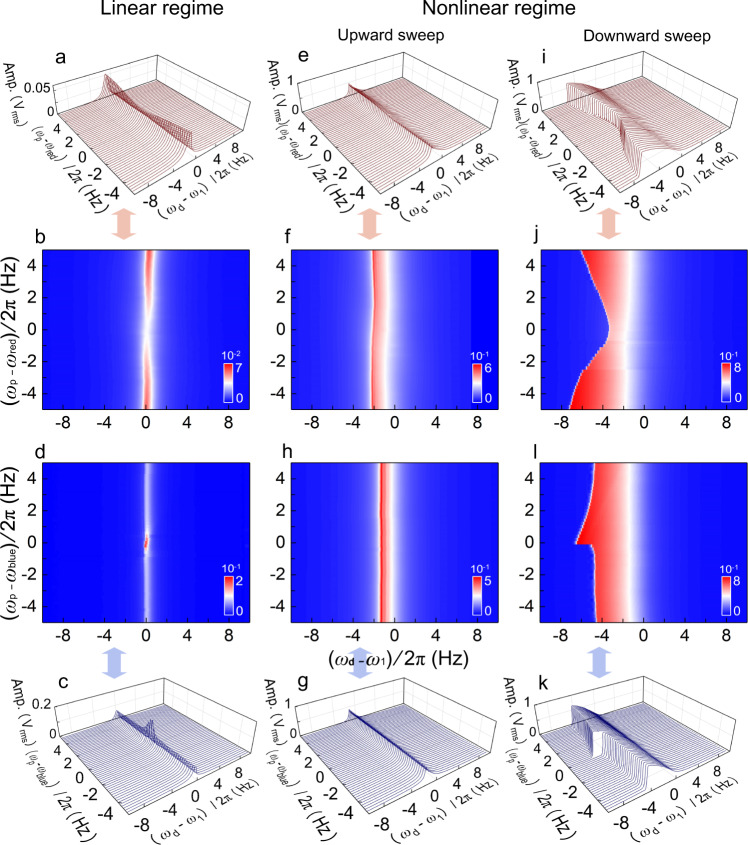
Linear versus nonlinear responses of the *ω*_1_ resonant mode parametrically coupled to the phonon cavity. **a** Resonant response of the *ω*_1_ mode in the linear regime under different pump frequency *ω*_p_, when pumping near the red sideband *ω*_red_ of the phonon-cavity. **b** 2D color plot of the same data as in **a**; same hereafter. The 2D color plots allow key features in the frequency domain (along both *ω*_d_ and *ω*_p_) to be better visualized, and enable efficient comparison to the theory plots in Fig. 3. **c**, **d** Resonant response in the linear regime when pumping near the blue sideband *ω*_blue_ of the phonon-cavity. **e**–**h** Nonlinear response of the *ω*_1_ mode with an upward frequency sweep, under different pump frequency *ω*_p_ near the red sideband *ω*_red_ (**e**, **f**) and blue sideband *ω*_blue_ (**g**, **h**), respectively. **i**–**l** Nonlinear response of the *ω*_1_ mode with a downward frequency sweep, under different pump frequency *ω*_p_ near the red sideband *ω*_red_ (**i**, **j**) and blue sideband *ω*_blue_ (**k**, **l**), respectively, showing clear modulation of the bi-stability frequency *ω*_b_ with distinct patterns. Experimental details are provided in “Methods” and SI 1.2, with additional data in SI 3.1.

Such process is experimentally manifested as the modulation in vibrational amplitude around *ω*_1_ when *ω*_p_ approaches either of the cavity sidebands. We first examine the parametric coupling to the phonon cavity with the resonant mode at *ω*_1_ excited in the linear regime (Fig. 2a–d), when scanning the excitation signal frequency *ω*_d_ around *ω*_1_ and the pump frequency *ω*_p_ around *ω*_red_ or *ω*_blue_. We find that when *ω*_p_ ≈ *ω*_red_ the resonant amplitude shows a clear dip (Fig. 2a, b), while near *ω*_p_ ≈ *ω*_blue_ it clearly peaks (Fig. 2c, d), showing excellent agreement with results in the literature^15,38^. This verifies that by using appropriate pump settings, our phonon cavity system can operate in the strong coupling regime with efficient energy transfer; and such phonon removal/creation processes can be efficiently controlled using the parametric pump.

We then investigate the phonon-cavity system with the resonant mode of interest excited into nonlinear vibration. As the driving voltage increases, the nonlinear resonant response develops bi-stability, and exhibits different behavior in upward (low to high) and downward (high to low) frequency sweeps (Fig. S3). We now scan *ω*_d_ around *ω*_1_ with both upward and downward sweeps, while also tuning *ω*_p_ around *ω*_red_ or *ω*_blue_. Both nonlinear responses show clear contrast with the linear case. In upward sweeps (Fig. 2e–h), the effect from the parametric pump is minimized, and the resonant responses remain largely unchanged as *ω*_p_ scans across both *ω*_red_ and *ω*_blue_, showing minimal energy exchange with the phonon-cavity.

In downward sweeps, however, the choice of the parametric pump frequency *ω*_p_ has very strong and distinct effects on the resonant response: when pumping in the vicinity of the red side band (Fig. 2i, j), the bi-stability frequency *ω*_b_ notably increases (moving towards *ω*_1_) as *ω*_p_ approaches *ω*_red_, producing a dip-like feature in the 2D color plot; when pumping around the blue side band (Fig. 2k, l), in contrast, the bi-stability frequency *ω*_b_ clearly decreases (moving away from *ω*_1_) with a sudden jump at *ω*_p_ ≈ *ω*_blue_, resulting in a spike-like feature in the 2D color plot (Fig. 2l). Interestingly, the effect of the parametric pump is clearly in the frequency domain, manifested as the modulation of *ω*_b_; in the displacement domain, however, for all *ω*_p_ settings the nonlinear resonance peak has been following the same frequency response curve (the curved slope of the shark-fin-like Duffing resonance peak) in the frequency down-sweeps, regardless the position of *ω*_b_. This is clearly visible in the 3D plots (Fig. 2i, k). Such behavior is in clear contrast to the linear case (Fig. 2a–d), where the parametric pump acts on the resonance of interest mostly in the displacement domain while causing little effect in the frequency domain.

To understand the unique behavior of the nonlinear response in our phonon-cavity system, we examine its equations of motion by introducing Duffing nonlinearity into this 2-DOF system^10,39^:

1 $$	{\ddot{x}}_{1}+{\omega }_{1}^{2}{x}_{1}=\, \varepsilon [-2{\gamma }_{1}{\dot{x}}_{1}-2({c}_{11}{x}_{1}+{c}_{12}{x}_{2})\cos ({\omega }_{{{{{{\rm{p}}}}}}}t)+{\alpha }_{1}{x}_{1}^{3} +{F}_{{{{{{\rm{d}}}}}}}\,\cos ({\omega }_{{{{{{\rm{d}}}}}}}t)]\\ 	 {\ddot{x}}_{2}+{\omega }_{2}^{2}{x}_{2}=\varepsilon [-2{\gamma }_{2}{\dot{x}}_{2} -2({c}_{21}{x}_{1}+{c}_{22}{x}_{2})\cos ({\omega }_{{{{{{\rm{p}}}}}}}t)]$$

here *x*_i_ and *ω*_i_ (*i* **=** 1, 2) are the displacement and natural frequency of the resonant modes, respectively. Specifically, *i* **=** 1 corresponds to the mode of interest (torsional mode, which we intentionally drive into nonlinear vibration, with *α*_1_ being the Duffing coefficient, and *F*_d_ cos(*ω*_d_*t*) being the harmonic driving force), and *i* **=** 2 corresponds to the phonon cavity (flexural mode, which remains undriven). The energy dissipation rates are given by *γ*_i_, and *c*_ij_ (*i*, *j* **=** 1, 2) give the intra-modal (*i* **=** *j*) and intermodal (*i* **≠** *j*) parametric coupling coefficients, with *ω*_p_ being the frequency of parametric pump. All these terms are normalized using the effective mass of the oscillator. *ε* is introduced as a scaling parameter, based on the assumption that all terms multiplied by *ε* are small compared with the terms on the left side of the equations. This assumption is true for steady-state vibration of high *Q* resonators, where the energy stored in the resonator (though constantly transforming between kinetic and potential energies) is much greater than dissipation or transfer between modes. This mathematical treatment allows us to apply multiple-scale approximation using the standard multidimensional Newton-Raphson algorithm^40^ (see SI 2.1 and 2.3–2.4 for details), from which we are able to numerically solve the above equations in the vicinity of the cavity sidebands (*ω*_p_ ≈ *ω*_red_ and *ω*_p_ ≈ *ω*_blue_), with the results shown in Fig. 3.

**Fig. 3:**
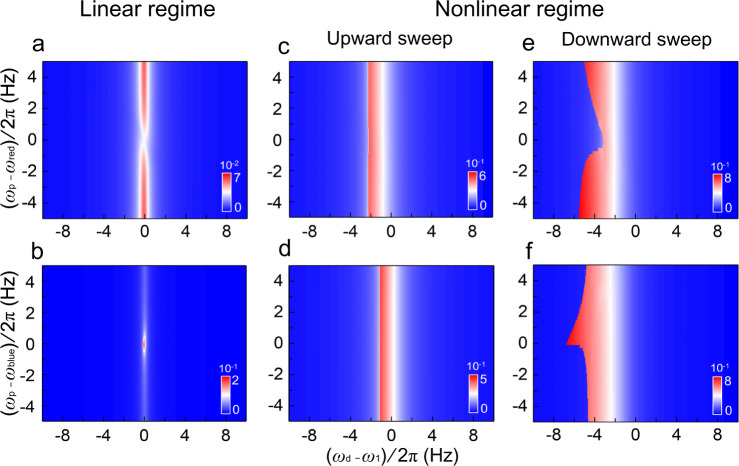
Numerical solutions to the phonon-cavity system operating in linear and nonlinear regimes. All results are presented in 2D color plots. **a**, **b** Calculated response of the *ω*_1_ mode in linear regime with pumping frequency *ω*_p_ near the (**a**) red sideband *ω*_red_ and **b** blue sideband *ω*_blue_ of the phonon-cavity. **c**, **d** Nonlinear response of the *ω*_1_ mode with an upward frequency sweep, with pump frequency *ω*_p_ near the red sideband *ω*_red_ (**c**) and blue sideband *ω*_blue_ (**d**), respectively. **e**, **f** Nonlinear response of the *ω*_1_ mode with a downward frequency sweep, with pump frequency *ω*_p_ near the red sideband *ω*_red_ (**e**) and blue sideband *ω*_blue_ (**f**), respectively, showing clear modulation of the bi-stability frequency *ω*_b_ with distinct patterns. Calculation and plot details are provided in “Methods” and SI 2.3–2.4, with additional data in SI 3.1.

The numerical results show very good agreement with the experimental data (for easy comparison, see Fig. 2 2D color plots) by reproducing all the key features. Specifically, in the downward sweep under nonlinear excitation, the calculation clearly produces in the 2D color plots the dip-like feature for *ω*_p_ near *ω*_red_, and the spike-like feature for *ω*_p_ near *ω*_blue_. Such unique behavior can be qualitatively understood, in a simplified picture, by considering the nonlinearity-induced amplitude bi-stability in the resonance mode, together with its parametric-pump-controlled coupling to the phonon-cavity: in downward frequency sweeps, the resonant response initially follows the upper branch among the two stable solutions from the softening Duffing equation, before jumping to the lower branch at *ω*_b_. When *ω*_p_ approaches *ω*_red_, the parametric pump causes phonon to be removed from the resonant mode (Fig. 1e), with the remaining ones insufficient to sustain the high-amplitude vibration on the upper branch, thus expedites the jumping to the lower branch (*ω*_b_ closer to *ω*_1_). In contrast, when *ω*_p_ ≈ *ω*_blue_, phonons are created in the resonant mode (Fig. 1f), which help in sustaining the vibration on the upper branch and delaying the jump (*ω*_b_ further away from *ω*_1_).

It is also interesting to analyze the observed phenomena by considering two different effects. First, with the sudden jump between its two stable branches, Duffing bi-stability produces a distinct relationship between the amplitude (*A*) and frequency (*f*) of a resonator, leading to an *A*-*f* relationship. Second, in phonon-cavity systems, the amplitude of a given mode is affected by the frequency difference between some other modes (such as the pump and the cavity modes), which gives rise to an *f*-*A* effect. By operating a phonon cavity device in a nonlinear regime, one can excite both effects, resulting in an *f*-*A*-*f* transduction, enabling signal amplification entirely within the frequency domain. While quantitative explanations for specific details (such as the asymmetry in *ω*_p_ for the dip-like and spike-like features) require more in-depth analyses and evaluation of multiple expressions derived from Eq. 1 (extensive details are offered in “Methods” and SI 2.3–2.4), the precise reproduction of experimental results allows us to further explore the unique nonlinear responses of our phonon-cavity system using numerical simulations.

**Fig. 4:**
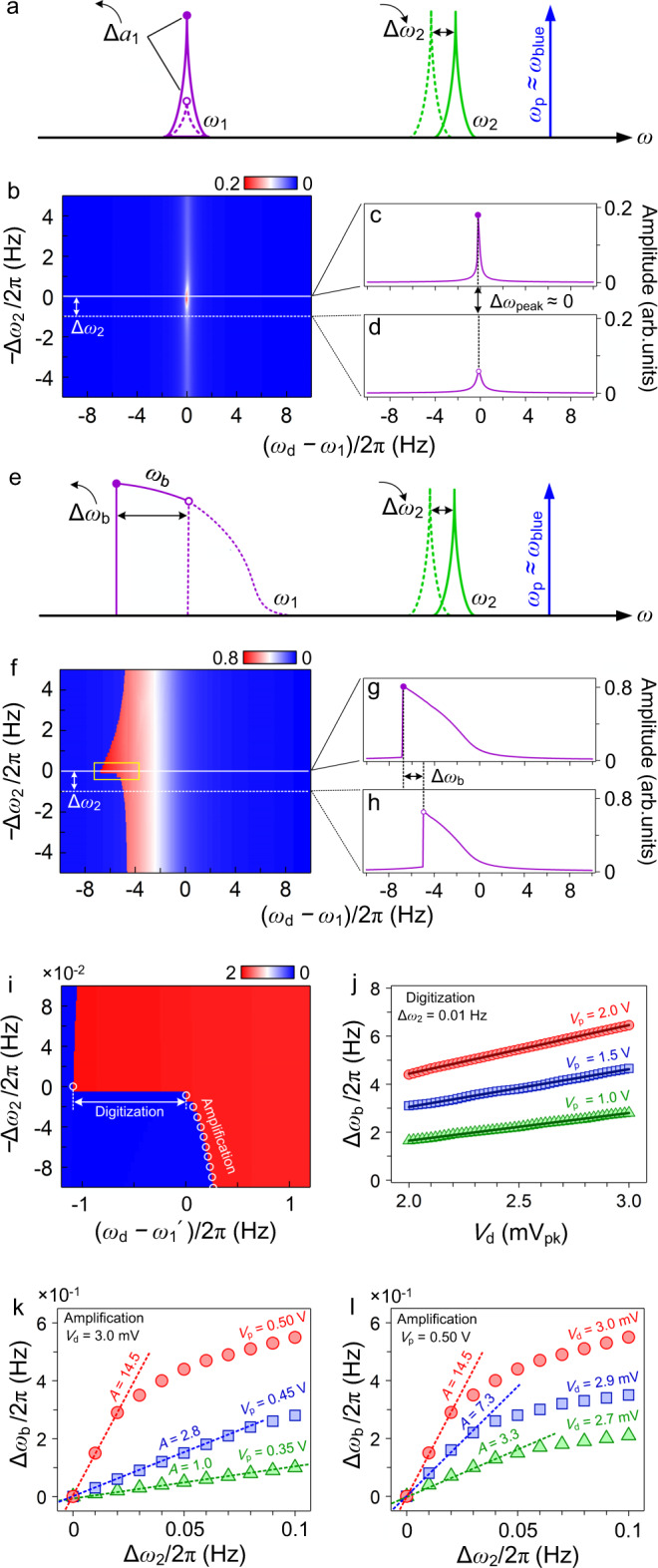
Signal transduction and amplification in the 2-DOF phonon cavity system. **a** Schematic illustration of the phonon-cavity’s response (purple signal) to a shift in the input signal (green) under linear vibration, when the pump signal (blue) is aligned with the cavity’s blue sideband. **b** 2D color plot of the phonon-cavity’s response to the input perturbation under linear vibration. **c**, **d** Line plots taken from different operation points in **b**, showing a negligible shift in resonance peak frequency *ω*_peak_. **e** Schematic illustration of the phonon-cavity’s response (purple signal) to a shift in the input signal (green) under nonlinear vibration, when the pump signal (blue) is aligned with the cavity’s blue sideband. **f** 2D color plot of the phonon-cavity’s response to the input perturbation under nonlinear vibration. **g**, **h** Line plots taken from different operation points in **f**, showing a significant shift in *ω*_b_. **i** A zoomed-in view of the boxed area in **f**, illustrating the regions used for the two frequency-domain operations. **j** The output shift in *ω*_b_ for a given input shift Δ*ω*_2_ **=** 0.01 Hz as functions of *V*_d_, under different *V*_p_ values. **k** The output shift in *ω*_b_ versus input shift in *ω*_2_ for different *V*_p_ values. Larger *V*_p_ corresponds to stronger parametric coupling. **l** The output shift in *ω*_b_ versus input shift in *ω*_2_ for different *V*_d_ values. Larger *V*_d_ corresponds to deeper nonlinearity. Plot details are provided in the “Methods” section.

We now show that such tuning of nonlinearity-mediated bi-stability, via parametric coupling to the phonon cavity, can be exploited to realize distinct functions in the frequency domain, offering unique capabilities for sensing and signal transduction. In resonant electromechanical transducers, typically, a change in an input physical quantity (e.g., pressure, acceleration, logic state) engenders a frequency shift, which is then measured or processed. In our devices, the phonon-cavity (resonant mode at *ω*_2_) can function as the front-end transducer, which converts the input signal to a shift in *ω*_2_ (green signals in Fig. 4a, e). When a pump signal is present at the cavity sideband (blue arrow), the input at *ω*_2_ can be coupled to the response at *ω*_1_. Here in Fig. 4 we discuss such processes with *ω*_p_ near the blue sideband (similar results are obtained for the red sideband, which are detailed in SI 3.2).

In the linear regime (Fig. 4a), the parametric coupling results in the mode at *ω*_1_ a change in its amplitude (purple signal), as the input change in *ω*_2_ is equivalent to a shift of the operation point (horizontal slices in Fig. 4b), with resulting traces presented in Fig. 4c, d for clear comparison. This is similar to a mode-localized resonant sensor^41^, which converts a shift in the frequency domain to a change in the displacement domain.

In the nonlinear regime (Fig. 4e), however, the input shift in *ω*_2_ is not transduced to the displacement domain; in contrast, it is amplified within the frequency domain, resulting in a much larger shift in *ω*_b_ (purple signal), as clearly demonstrated in both experiment (Fig. 2l) and theory (Fig. 3f). Here, the key is to leverage the unique spike-like feature by operating near its sharp edge (horizontal slices in Fig. 4f) in order to achieve augmented output in the frequency domain (Fig. 4g, h). Given the unique response of *ω*_b_ in this region, we show that two intriguing sensing-related functions can be realized: digitization and amplification (Fig. 4i, which zooms into the boxed area of Fig. 4f).

In the first scenario, we utilize the horizontal red-blue boundary in Fig. 4i at *ω*_p_ **=** *ω*_blue_, indicated by the double-headed arrow. A small shift in *ω*_2_ (0.01 Hz, for example, as used for Fig. 4j) can cause the operating point to cross this sharp edge, resulting in a discrete and sizable change (several Hz, as found in Fig. 4j) in the output *ω*_b_ value. The magnitude of discretization (shift in output *ω*_b_) can be continuously and smoothly controlled by adjusting the pump strength *V*_p_ and the degree of nonlinearity (varying *V*_d_), as shown in Fig. 4j. Such digitization of the signal could be potentially useful for sensing applications, by determining whether the signal exceeds a given threshold.

In the other scenario, we operate along the sloped red-blue boundary (*ω*_p_ slightly below *ω*_blue_), outlined by the small circles in Fig. 4i. In this region, a change in *ω*_2_ from the input engenders a continuous and amplified output (change in *ω*_b_), for which we define the gain *A* as:

2 $$A=\frac{\Delta {{{{{\rm{Output}}}}}}}{\Delta {{{{{\rm{Input}}}}}}}=\frac{\Delta {\omega }_{{{{{{\rm{b}}}}}}}}{\Delta {\omega }_{2}}$$

By exploring the parameter space, we observe a number of behaviors for this amplification effect. First, the gain (slope of the data in Fig. 4k, l) can be controlled by both the pump strength *V*_p_ and the degree of nonlinearity (adjusting *V*_d_). We find that deeper nonlinear vibration or stronger parametric coupling can produce larger amplification (see SI 3.3 for additional details), even exceeding one order of magnitude (red data series). Second, there is a trade-off between gain and linear dynamic range: a larger gain is associated with a smaller dynamic range. By carefully adjusting *V*_d_ and *V*_p_, one can choose the optimal operating condition.

While frequency-sweep measurements are used to explore the exquisite nonlinear dynamics in such phonon-cavity systems, towards device applications a more efficient readout scheme is required. Here we demonstrate both amplification and digitization operations with fast readout using a phase-locked loop (PLL).

The PLL set-up is shown in Fig. S8. A DC bias *V*_f_ is applied (blue lines) on the electrodes to shift the frequency of mode 2 (*ω*_2_). In practice, the shift of *ω*_2_ can be caused by external factors such as temperature, pressure, acceleration, and angular velocity. Here, in order to demonstrate the feasibility of the proposed functions, we use the negative stiffness due to electrostatic force to simulate the frequency shift of mode 2 due to such external factors. To minimize the effect from environment variables, the MEMS resonator is measured in a vacuum with a constant temperature of 30 °C. The frequency output of mode 1 (*ω*_b_) is monitored (purple lines) using PLL in addition to the frequency sweep measurements (frequency response analysis (FRA)).

**Fig. 5:**
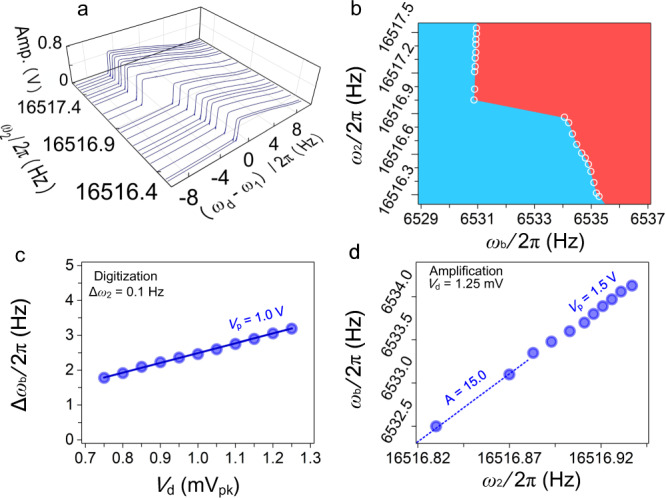
Experimental demonstration of signal transduction and amplification using the nonlinear phonon-cavity system at the blue pump. **a** The frequency response curves as functions of *ω*_2_ in downward frequency sweep measurements. **b** Fast measurement of *ω*_b_ as functions of *ω*_2_. The white circles are the PLL measured data, and the solid colors on both sides offer visual cues to the eye. **c** The output shift in *ω*_b_ as functions of *ω*_2_ in the digitization region measured by PLL. **d** The output *ω*_b_ as functions of *ω*_2_ in the amplification region measured by PLL.

The experimental results for the phonon-cavity system at blue and red pumps are shown in Fig. 5 and Fig. S9, respectively. Here we use the blue pump case as an example. Following the numerical simulation results in Fig. 4, we first perform a frequency sweep experiment (Fig. 5a) and verify the phonon cavity response as predicted in Fig. 4i. We then use the DC voltage *V*_f_ to control *ω*_2_, and monitor the frequency output *ω*_b_ using PLL. The PLL data (Fig. 5b) clearly captures all the *ω*_b_ values in Fig. 5a, again demonstrating the nonlinear behavior predicted in Fig. 4i. We note that with PLL *ω*_b_ can be measured much faster than in frequency sweeps, and the bandwidth of the measurement system can be optimized by setting appropriate PID parameters and reference phase of mode 1.

To verify the signal sensing enchancement functions, we operate the phonon cavity system in corresponding parameter spaces. Figure 5c, d present the PLL experimental results of the digitization and amplification operations respectively, and both show good agreement with the anticipated behavior shown in Fig. 4j, k/l. We note that given the different operating parameters (such as *V*_d_ and *V*_p_) used in simulation and experiments, the results may not exactly agree quantitatively, but qualitatively all key features and behaviors are successfully reproduced experimentally.

Similarly, the experimental results for the red pump case (Fig. S9) agree well with the numerical predictions (Fig. S6). While standalone instruments are used to demonstrate the PLL measurement scheme here, in practice with known design parameters such PLL component can be efficiently realized in circuit designs, thus facilitating a high level of integration of such frequency-shift-based sensors.

These unique nonlinearity-mediated frequency-shift functions can be exploited for constructing phonon-cavity-based sensors and transducers. It is worth noting that operating devices in the frequency domain have a number of advantages, as “time and frequency are the most accurately measurable of all physical quantities”^42^. Our results show that, by harnessing nonlinearity in phonon-cavity system, intriguing opportunities emerge for high-performance frequency-shift-based sensors and transducers^43–50^. Specifically, compared with MEMS sensors and transducers using single-DOF nonlinear resonators^51^ or multiple coupled resonators^52,53^, the nonlinear phonon-cavity system in this work could potentially offer greater responsivity, additional control, smaller device footprint, and simplified system design, thus resulting in improved performance in MEMS-based sensing and signal transduction applications.

## Methods

### Device design

The electromechanical structure is designed to facilitate coherent energy transfer between the two resonant modes via parametric pump-controlled intermodal coupling. A three-segment beam is used to couple the two masses together, facilitating intermodal coupling structurally. A large mass area and the small capacitive gap in the device design make it easier for the resonator to exhibit nonlinearity, without requiring excessive amplitude. The design of multiple groups of electrodes under the resonator allows us to efficiently adjust the frequency of the different modes and implement dynamic coupling at the same time. The quality factor of the resonator can be effectively controlled by the vacuum packaging process.

### Device fabrication

The die embedding the vacuum-sealed MEMS resonators is electrically packaged in a ceramic leadless chip carrier with a pressure of 0.1 Pa. The structure layer is 40-μm-Si and the electrode layer is 6-μm-Si. The gap between the structure layer and the electrode layer is 2 μm. The lateral size of the resonator is about 3 mm × 3 mm. The insulating layer underneath the electrode layer is 2-μm-SiO_2_. The device is operated under room temperature. The device structure, including its cross-section, and the fabrication process are detailed in Fig. S1.

### Experimental set-up

A number of instruments are used in the measurement. The bias voltages are generated by a low noise voltage source (ITECH IT6233). The drive and pump signals are provided by a two-channel lock-in amplifier (Zurich Instruments HF2LI). The response motion of the resonator is detected by a capacitance–voltage (C/V) converting scheme based on a charge amplifier and measured by the lock-in amplifier. Detailed measurement setup and wiring diagram are available in SI 1.2.

### Resonance measurements

The linear and nonlinear responses of both resonant modes are first characterized in the absence of the parametric pump. The measurement condition for the dataset shown in Fig. 1 is as follows: the driving voltage of mode 1 and mode 2 are *V*_d_ = 0.2, 0.4, 0.6, 0.8, 1.0 mV_pk_ and *V*_d_ = 1, 2, 3, 4, 5 mV_pk_, respectively. The results in (c) and (d) are both measured with a frequency downward sweep.

The coherent energy transfer in the phonon cavity is characterized by scanning the parametric pump frequency near either of the cavity sidebands. The measurement and plotting details for Fig. 2 are as follows:

In the linear regime when *ω*_p_ ≈ *ω*_red_ (a, b), the device is measured with *V*_d_ = 0.05 mV_pk_, *V*_p_ = 5.0 V_pk_, and plotted using *ω*_1_ = 2*π* × 6886.0 Hz, *ω*_red_ = 2*π* × 9592.0 Hz. In the linear regime when *ω*_p_ ≈ *ω*_blue_ (c, d), the device is measured with *V*_d_ = 0.05 mV_pk_, *V*_p_ = 2.0 V_pk_, and plotted using *ω*_1_ = 2*π* × 6958.0 Hz, *ω*_blue_ = 2*π* × 23,582.0 Hz.

In the nonlinear regime upward sweep, when *ω*_p_ ≈ *ω*_red_ (e, f), the device is measured with *V*_d_ = 1.5 mV_pk_, *V*_p_ = 5.0 V_pk_. and plotted using *ω*_1_ = 2*π* × 6886.0 Hz, *ω*_red_ = 2*π* × 9592.5 Hz. When *ω*_p_ ≈ *ω*_blue_ (h, g), the device is measured with *V*_d_ = 1.0 mV_pk_, *V*_p_ = 1.5 V_pk_. and plotted using *ω*_1_ = 2*π* × 6958.0 Hz, *ω*_blue_ = 2*π* × 23,575.0 Hz.

In the nonlinear regime downward sweep, when *ω*_p_ ≈ *ω*_red_ (i, j), the device is measured with *V*_d_ = 1.5 mV_pk_, *V*_p_ = 5.0 V_pk_. and plotted using *ω*_1_ = 2*π* × 6886.0 Hz, *ω*_red_ = 2*π* × 9592.5 Hz. When *ω*_p_ ≈ *ω*_blue_ (k, l), the device is measured with *V*_d_ = 1.0 mV_pk_, *V*_p_ = 1.5 V_pk_. and plotted using *ω*_1_ = 2*π* × 6958.0 Hz, *ω*_blue_ = 2*π* × 23,575.0 Hz.

In all measurements in Fig. 2, the frequency scanning steps are the same: ∆*ω*_d_ = 2*π* × 0.05 Hz, ∆*ω*_p_ = 2*π* × 0.2 Hz. Note that the plot center frequencies are slightly adjusted for each panel for easy comparison across the different measurement conditions, in order to best illustrate the key findings. Similarly, the driving and pumping strength for the data presented in each panel are also chosen to best illustrate the key findings.

### Numerical analysis

The numerical codes are constructed using C language. The numerical settings for producing the data in Fig. 3 are as follows:

In the linear regime when *ω*_p_ ≈ *ω*_red_ (a), the device response is calculated with *V*_d_ = 0.05 mV_pk_, *V*_p_ = 5.0 V_pk_, and plotted using *ω*_1_ = 2*π* × 6886.0 Hz, *ω*_red_ = 2*π* × 9592.0 Hz. In the linear regime when *ω*_p_ ≈ *ω*_blue_ (b), the device response is calculated with *V*_d_ = 0.05 mV_pk_, *V*_p_ = 2.0 V_pk_, and plotted using *ω*_1_ = 2*π* × 6958.0 Hz, *ω*_blue_ = 2*π* × 23,582.0 Hz.

In the nonlinear regime upward sweep, when *ω*_p_ ≈ *ω*_red_ (c), the device response is calculated with *V*_d_ = 1.5 mV_pk_, *V*_p_ = 5.0 V_pk_, and plotted using *ω*_1_ = 2*π* × 6886.0 Hz, *ω*_red_ = 2*π* × 9592.5 Hz. When *ω*_p_ ≈ *ω*_blue_ (d), the device response is calculated with *V*_d_ = 1.0 mV_pk_, *V*_*p*_ = 2.0 V_pk_, and plotted using *ω*_1_ = 2*π* × 6958.0 Hz, *ω*_blue_ = 2*π* × 23,575.0 Hz.

In the nonlinear regime downward sweep, when *ω*_p_ ≈ *ω*_red_ (e), the device response is calculated with *V*_d_ = 1.5 mV_pk_, *V*_p_ = 5.0 V_pk_. and plotted using *ω*_1_ = 2*π* × 6886.0 Hz, *ω*_red_ = 2*π* × 9592.5 Hz. When *ω*_p_ ≈ *ω*_blue_ (f), the device response is calculated with *V*_d_ = 1.0 mV_pk_, *V*_p_ = 2.0 V_pk_, and plotted using *ω*_1_ = 2*π* × 6958.0 Hz, *ω*_blue_ = 2*π* × 23,575.0 Hz.

In all calculations presented in Fig. 3, the frequency scanning steps are the same: ∆*ω*_d_ = 2*π* × 0.05 Hz, ∆*ω*_p_ = 2*π* × 0.2 Hz. Note that the plot center frequencies are slightly adjusted for each panel for easy comparison across the different measurement conditions, in order to best illustrate the key findings. Similarly, the driving and pumping strength for the data presented in each panel are also chosen to best illustrate the key findings.

### Experimental demonstration of digitization and amplification functions using PLL

The numerical settings for producing the data in Fig. 4 are as follows:

In the linear operation (b), *V*_d_ = 0.05 mV_pk_, *V*_p_ = 2.0 V_pk_, *ω*_1_ = 2*π* × 6958.0 Hz, and *ω*_p_ = 2*π* × 23,582.0 Hz. The frequency steps are ∆*ω*_d_ = 2*π* × 0.05 Hz, and ∆*ω*_2_ = 2*π* × 0.2 Hz. The line plots (c, d) are taken from data in (b) with ∆*ω*_2_ = 0 (c) and ∆*ω*_2_ = 2*π* × 1.0 Hz (d).

In the nonlinear operation with downward sweep (f), *V*_d_ = 1.0 mV_pk_, *V*_p_ = 2.0 V_pk_, *ω*_1_ = 2*π* × 6958.0 Hz, and *ω*_p_ = 2*π* × 23,575.0 Hz. The frequency steps are ∆*ω*_d_ = 2*π* × 0.05 Hz, and ∆*ω*_2_ = 2*π* × 0.2 Hz. The line plots (g, h) taken from data in (d) when ∆*ω*_2_ = 0 (g) and ∆*ω*_2_ = 2*π* × 1.0 Hz (h).

In the zoom-in plot (i), *V*_d_ = 3.0 mV_pk_, *V*_p_ = 0.6 V_pk_, *ω*_1_ = 2*π* × 6929.52 Hz, and *ω*_p_ = 2*π* × 23,552.51 Hz. The frequency steps are ∆*ω*_d_ = 2*π* × 0.01 Hz, ∆*ω*_2_ = 2*π* × 0.01 Hz.

The data of digitization in (j) are calculated for *V*_p_ = 1.0, 1.5, 2.0 V_pk_ when *V*_d_ is scanned from 2.0 mV_pk_ to 3.0 mV_pk_. The step of *V*_d_ is 0.1 mV_pk_.

The data of amplification in (k) are calculated for *V*_d_ is 3.0 mV_pk_ and *V*_p_ = 0.35, 0.45, 0.50 V_pk_. The step of ∆*ω*_2_ is 2*π* × 0.01 Hz.

The data of amplification in (l) are calculated for *V*_p_ is 0.50 V_pk_ and *V*_d_ = 2.7, 2.9, 3.0 mV_pk_. The step of ∆*ω*_2_ is 2*π* × 0.01 Hz.

### Experimental demonstration of digitization and amplification functions using PLL

The measurement settings for producing Fig. 5 are as follows:

In the nonlinear operation at blue pump with downward sweep (a), *V*_d_ = 1.25 mV_pk_, *V*_p_ = 1.5 V_pk_, *ω*_1_ = 2π × 6538.0 Hz, and *ω*_p_ = 2*π* × 23,048.0 Hz. The steps are ∆*ω*_d_ = 2*π* × 0.02 Hz, and ∆*V*_f_ = 0.05 V_pk_.

When using PLL to test the phonon-cavity system at blue pump (b), *V*_d_ = 1.25 mV_pk_, *V*_p_ = 1.5 V_pk_, and *ω*_p_ = 2π × 23,048.0 Hz. *V*_f_ is scanned from 0 to 1 V_pk_ and the step of *V*_f_ is 0.05 V_pk_.

The experiment of digitization at blue pump in (c), *V*_p_ = 1.5 V_pk_ when *V*_d_ is scanned from 0.75 mV_pk_ to 1.25 mV_pk_. The step of *V*_d_ is 0.05 mV_pk_.

The experiment of amplification at blue pump in (d), *V*_d_ = 1.25 mV_pk_, *V*_p_ = 1.5 V_pk_, and *ω*_p_ = 2*π* × 23,048.0 Hz. *V*_f_ is scanned from 0.445 to 0.495 _pk_ and the step of *V*_f_ is 5 mV_pk_.

### Frequency noise of the phonon-cavity system

The experiment result of frequency noise is shown in Fig. S10. Here, we measure the frequency noise of input signal *ω*_2_ and output signal *ω*_b_ under different operation conditions: when there is no parametric pump when there is a blue pump and when there is a red pump applied to the phonon-cavity system.

The time-domain data is shown in Fig. S10a, and the calculated Allan deviation *σ* is shown in Fig. S10b. According to the fitting results, the frequency random walk and instability are calculated and shown in Table S2. The results show that the signal transduction through the phonon-cavity system does not increase the frequency noise. Interestingly, it shows that when operating under a blue pump, the noise performance can even improve.

## Supplementary information

Supplementary Information

Peer Review File

## Data Availability

The datasets generated during and/or analyzed during the current study are available from the corresponding authors on reasonable request.
