# Supplementary material for: Nonlinearity-mediated digitization and amplification in electromechanical phonon-cavity systems
Source: Nat Commun. 2022 Apr 29;13:2352. doi: 10.1038/s41467-022-29995-x (PMC9054851; doi:10.1038/s41467-022-29995-x)
Supplement: Supplementary file 1 — Supplementary Information [file 41467_2022_29995_MOESM1_ESM.pdf]

Supplementary Information

Nonlinearity-Mediated Digitization and Amplification in Electromechanical Phonon-Cavity Systems

Tongqiao Miao[1], Xin Zhou[1], Xuezhong Wu[1,4,5], Qingsong Li[1], Zhanqiang Hou[1], Xiaoping Hu[1], Zenghui Wang[2,3]*, and Dingbang Xiao[1,4,5]*

[1]College of Intelligence Science, National University of Defense Technology, Changsha 410073, China

[2]Institute of Fundamental and Frontier Sciences, University of Electronic Science and Technology of China, Chengdu, 610054, China

[3]State Key Laboratory of Electronic Thin Films and Integrated Devices, University of Electronic Science and Technology of China, Chengdu 610054, China

[4]Laboratory of Science and Technology on Integrated Logistics Support, National University of Defense Technology, Changsha 410073, China

[5]MEMS Engineering Center of Hunan, Changsha 410073, China.

*corresponding authors: zenghui.wang@uestc.edu.cn, dingbangxiao@nudt.edu.cn

1. EXPERIMENTAL SETUP

1.1 Fabrication of the MEMS device

The fabrication process of the MEMS device is shown in Fig. S1. The process is illustrated in 8 steps.

(1) We start with an SOI wafer, with 6 μm device layer and 2 μm box layer. The resistivity of the device layer is about 0.005 ohm-cm.

(2) First, the wafer bonding regions (later to the resonator body above) are patterned on the Si device layer.

(3) The electrode regions are then patterned on the device layer, with height reduced (2 μm) to facilitate wafer bonding.

(4) We perform Si-Si bonding to add another layer of Si (40 μm) on top (for making resonator body).

(5) Al metal (500 nm) is patterned on top to define the bonding pads.

(6) Top layer Si is patterned to define the resonator body.

(7) The Si cap is prepared with getter (Ti) for wafer scale packaging.

(8) The cap wafer is bonded to the device wafer using glass frit bonding.

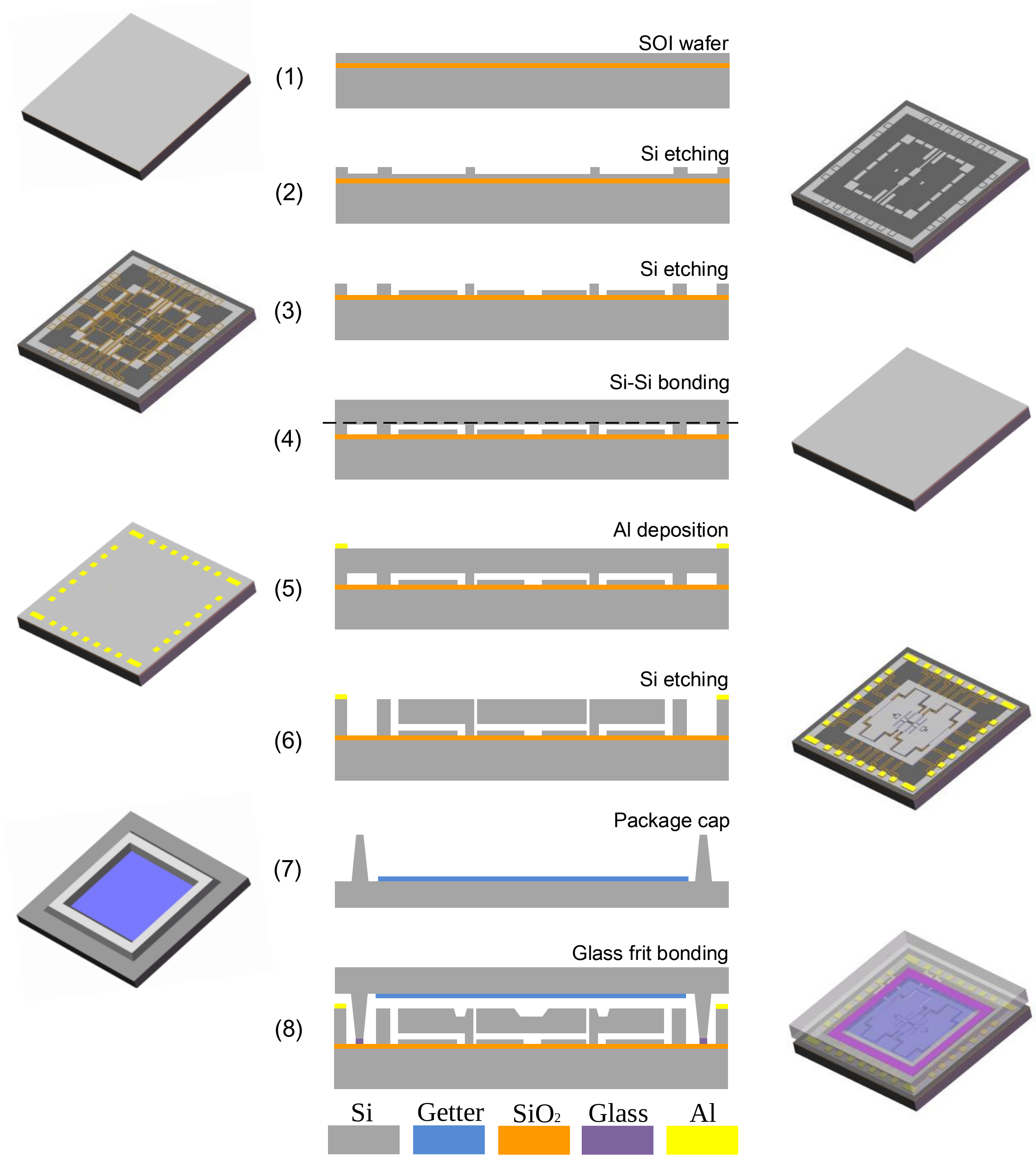

FIG. S1. The fabrication process of the MEMS device. (1) Preparing a SOI wafer. (2) Etching Si for bonding regions. (3) Etching Si for electrode patterns. (4) Si-Si bonding. (5) Al deposition for pad metallization. (6) Etching Si for resonator structure. (7) Preparing the package cap with getter. (8) Glass frit bonding for the final device.

1.2 Measurement scheme

The measurement system is shown in Fig. S2. There are two groups of silicon electrodes beneath the resonator body, illustrated with purple and green colors for clarity. The purple electrodes are used to drive and detect the torsional mode (mode 1), and the green ones are used to drive and detect the flexural mode (mode 2). V_d is the AC output signal from the lock-in used to drive mode 1 and mode 2, respectively, and V_p is the pump signal. A DC bias V_b is applied on the resonator body to facilitate the capacitive readout, and is maintained at 2.5 V throughout all measurements. The bias voltages are supplied by a low noise voltage source (ITECH IT6233). The drive and pump signals are supplied by a two-channel lock-in amplifier (Zurich Instruments HF2LI).

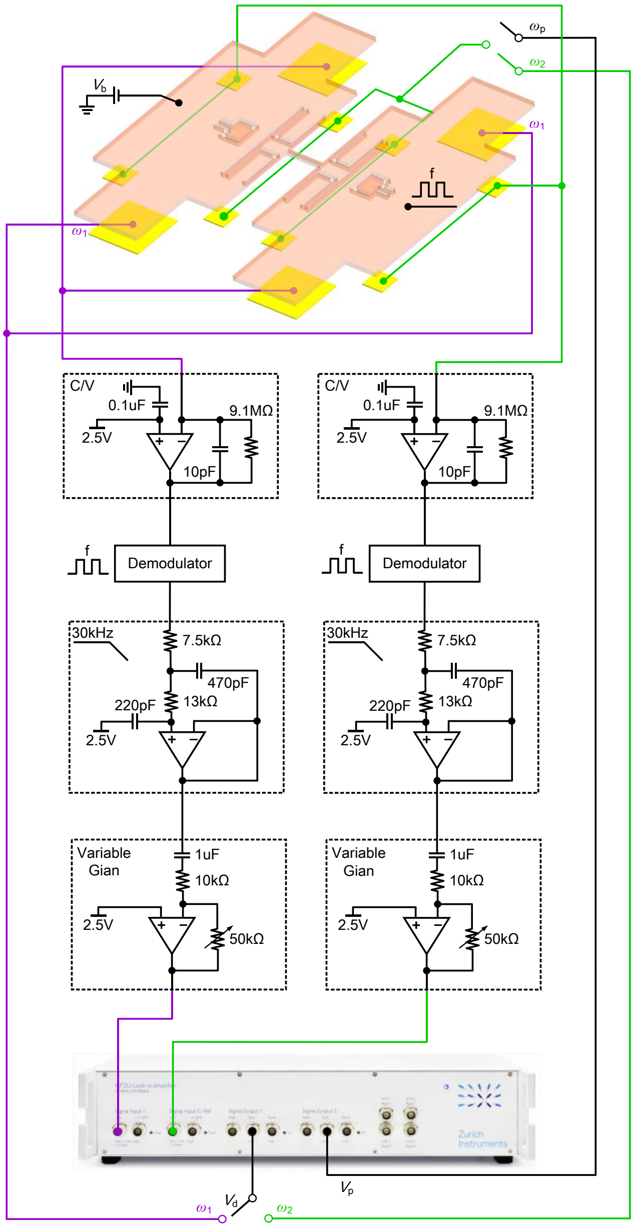

FIG. S2. Schematic diagram of electrical measurements of the phonon cavity.

In order to suppress low frequency noise, a square wave carrier signal (f = 1 MHz) is also applied on the silicon structure to modulated displacement signal. The response motion of the resonator is detected using a capacitance–voltage (C/V) transduction scheme based on charge amplification [31], and measured by the lock-in amplifier. Also, the demodulator with the demodulation signal of f and the filter with the cut-off frequency of 30 KHz are designed to separate the displacement signal. The variable gain module is designed for easily adjusting the gain of response.

1.3 Measurement scheme

As the vibrational amplitude increases, resonators with Duffing nonlinearity will exhibit two stable branches in the solution to its equation of motion (bifurcation occurs), as show in Fig. S3. When measuring such nonlinear responses, depending on the frequency sweep direction, the Duffing curve can take either the upper or lower branch, before jumping to the other branch. The jumping between the two branches occurs at specific frequencies, which also depends on the sweeping direction. The frequency at which such jumping takes place is defined as ω_b. Specifically, as in most of the measurements we are examining the downward frequency sweep, in this work ω_b refers to the downward jump during the downward frequency sweep (as noted in Fig. S3) unless otherwise specified.

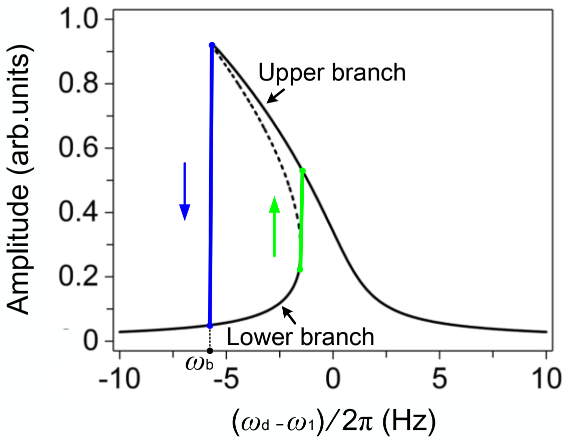

FIG. S3. The schematic diagram of nonlinear vibration response, and the definition of ω_b.

2. THEORETICAL ANALYSIS

2.1 Single-mode nonlinear oscillator

The theoretical analysis of dynamical coupling can be started by first solving the nonlinear vibration of mode 1. Here, we first deal with a single-mode Duffing oscillator with cubic nonlinear term:

$$\ddot{x}_1 + \omega_1^2 x_1 = \varepsilon\left[-2\gamma_1 \dot{x}_1 + \alpha_1 x_1^3 + F_\mathrm{d}\cos\left(\omega_\mathrm{d} t\right)\right] \tag{S1}$$

where x_1 is the displacement, ω_1 is the natural frequency, γ_1 is the energy dissipation rate, α_1 is the Duffing cubic coefficient, which is mainly dominated by capacitive nonlinearity. F_d is the amplitude of harmonic driving force. Note that all these terms are normalized by the effective mass of the oscillator. The factor ε is formally introduced as a small and dimensionless parameter, which can be used to facilitate the approximate solution [40].

We can apply the method of Multiple-scale approximation [40] to get the stationary solution of Eq. (S1). The method assumes that the solution depends on time through two auxiliary variables, $T_0 \equiv t$ and $T_1 = \varepsilon t$, representing the fast and slow scales respectively. On the one hand, the fast oscillations have frequencies of the same order as ω_1 and ω_d. On the other hand, the oscillation amplitudes and phases change much more slowly. Therefore, the solution can be expanded as:

$$\begin{aligned} x_1\left(T_0,T_1\right) &= x_{10}\left(T_0,T_1\right) + \varepsilon x_{11}\left(T_0,T_1\right) \\ x_{10} &= \frac{1}{2} a_1\left(T_1\right) e^{i\left[\omega_1 T_0 + \beta_1\left(T_1\right)\right]} + cc \end{aligned} \tag{S2}$$

where a_1 and β_1 are real numbers, which depend on T_1 only; cc stands for the complex conjugate of the preceding terms. By substituting Eq. (S2) into Eq. (S1) and equating the coefficient of ε on both sides, we obtain:

$$D_0^2 x_{11} + \omega_1^2 x_{11} = -2D_0 D_1 x_{10} - 2\gamma_1 D_0 x_{10} + \alpha_1 x_{10}^3 + F_\mathrm{d}\cos\left(\omega_\mathrm{d} t\right) \tag{S3}$$

where the derivative operators are defined as:

$$\begin{cases} \dfrac{d}{dt} = \dfrac{\partial}{\partial T_0}\dfrac{dT_0}{dt} + \dfrac{\partial}{\partial T_1}\dfrac{dT_1}{dt} = D_0 + \varepsilon D_1 \\ \dfrac{d^2}{dt^2} = D_0^2 + 2\varepsilon D_0 D_1 + \varepsilon^2 D_1^2 \end{cases} \tag{S4}$$

We introduce the detuning parameters σ_d to express the harmonic excitation:

$$\omega_\mathrm{d} = \omega_1 + \varepsilon\sigma_\mathrm{d} \tag{S5}$$

By substituting Eq. (S5) into Eq. (S3) and eliminating secular terms from the particular solution of Eq. (S3), we obtain:

$$-i\omega_1\left(a_1'+\gamma_1 a_1\right)+\omega_1 a_1\beta_1'+\frac{3}{8}\alpha_1 a_1^3+\frac{1}{2}F_\mathrm{d}e^{i(\sigma_\mathrm{d}T_1-\beta_1)}=0 \tag{S6}$$

Then we separate the result into its real and imaginary parts and obtain:

$$\begin{aligned} &8\omega_1 a_1\beta_1'+3\alpha_1 a_1^3+4F_\mathrm{d}\cos\left(\sigma_\mathrm{d}T_1-\beta_1\right)=0\\ &2\omega_1 a_1'+2\gamma_1\omega_1 a_1-F_\mathrm{d}\sin\left(\sigma_\mathrm{d}T_1-\beta_1\right)=0 \end{aligned} \tag{S7}$$

Eq. (S7) can be transformed into an autonomous system (*i.e.*, one in which T_1 does not appear explicitly) by letting:

$$\theta_1=\sigma_\mathrm{d}T_1-\beta_1 \tag{S8}$$

and steady-state motion takes place when:

$$a_1'=\theta_1'=0 \tag{S9}$$

By substituting Eq. (S9) into Eq. (S7), we obtain:

$$a_1^2=\frac{F_\mathrm{d}^2}{4\omega_1^2\left[\gamma_1^2+\left(\sigma_\mathrm{d}-\kappa a_1^2\right)^2\right]} \tag{S10}$$

which is the well-known frequency response of Duffing resonator, with $\kappa = -3\alpha_1/8\omega_1$. From Eq. (S10), it can be found that the maximum steady amplitude of vibration can be obtained when:

$$\sigma_\mathrm{d}=\kappa a_1^2 \tag{S11}$$

which shows that the resonant frequency of single-mode Duffing oscillator is relevant to the amplitude of vibration a_1 because of the cubic nonlinear term.

2.2 Duffing cubic coefficient

The term α_1 in Eq. (S1) is the Duffing cubic coefficient, which in our device is dominated by the capacitive nonlinearity. This assumption is valid for our device, because mode 1 (the torsional mode) has negligible mechanical nonlinearity for the relevant vibration amplitudes [38]. Here we analyze the capacitive nonlinearity in our device.

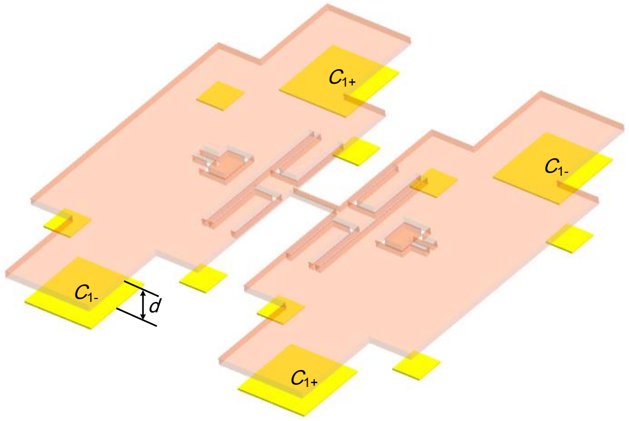

FIG. S4. Diagram of Duffing nonlinearity analysis.

The differential configuration is used to drive the motion of the resonator in the experiment, which means four electrodes of mode 1 (C_{1+}, C_{1-}, as illustrated in Fig. S4) are all used to excite and detect the resonator. We denote d as the spacing between the electrode and the resonator body, and the driving voltage (V_{d+}, V_{d-}) on the two pairs of electrodes (C_{1+}, C_{1-}), respectively, can be expressed as:

$$\begin{aligned} V_{\mathrm{d}+} &= V_{\mathrm{dc}} + V_{\mathrm{ac1}}\cos(\omega_{\mathrm{d}} t) \\ V_{\mathrm{d}-} &= V_{\mathrm{dc}} - V_{\mathrm{ac1}}\cos(\omega_{\mathrm{d}} t) \end{aligned} \tag{S12}$$

Note that in order to clearly illustrate the effect of DC and AC components, here we use V_{dc} to represent the V_b in Fig. S1, and use V_{ac} to represent the V_d in Fig. S1. The electrostatic force (F_{d+}, F_{d-}) on the two pairs of electrodes (C_{1+}, C_{1-}), respectively, can then be expressed as：

$$\begin{aligned} F_{\mathrm{d}+} &= \frac{1}{2}\times 2\frac{\mathrm{d}C_{1+}}{\mathrm{d}d}\left[V_{\mathrm{dc}} + V_{\mathrm{ac1}}\cos(\omega_{\mathrm{d}} t)\right]^2 \\ F_{\mathrm{d}-} &= \frac{1}{2}\times 2\frac{\mathrm{d}C_{1-}}{\mathrm{d}d}\left[V_{\mathrm{dc}} - V_{\mathrm{ac1}}\cos(\omega_{\mathrm{d}} t)\right]^2 \end{aligned} \tag{S13}$$

Assuming that the initial gap and capacitance of single capacitor is d and C_1, when a small change Δd occurs ($\Delta d << d$), Eq. (S13) can be expanded based on the Taylor expansion:

$$\begin{cases} F_{\mathrm{d}+} = \dfrac{C_1}{d}(V_{\mathrm{dc}}^2 + 2V_{\mathrm{ac1}}V_{\mathrm{dc}}\cos\omega_{\mathrm{d}}t + V_{\mathrm{ac1}}^2\cos^2\omega_{\mathrm{d}}t)\left[1+\dfrac{2}{d}\Delta d+\dfrac{3}{d^2}\Delta d^2+\dfrac{4}{d^3}\Delta d^3+o(\Delta d^4)\right] \\ F_{\mathrm{d}-} = \dfrac{C_1}{d}(V_{\mathrm{dc}}^2 - 2V_{\mathrm{ac1}}V_{\mathrm{dc}}\cos\omega_{\mathrm{d}}t + V_{\mathrm{ac1}}^2\cos^2\omega_{\mathrm{d}}t)\left[1-\dfrac{2}{d}\Delta d+\dfrac{3}{d^2}\Delta d^2-\dfrac{4}{d^3}\Delta d^3+o(\Delta d^4)\right] \end{cases} \quad \text{(S14)}$$

As $V_{\mathrm{ac1}} << V_{\mathrm{dc}}$, the nonlinear terms caused by V_{ac1} are ignored:

$$F_{\mathrm{dc}} = F_{\mathrm{d}+} - F_{\mathrm{d}-} = \frac{4C_1}{d}V_{\mathrm{dc}}^2(\frac{1}{d}\Delta d + \frac{2}{d^3}\Delta d^3) \quad \text{(S15)}$$

According to the movement process of mode 1 in Eq. (S1), Δd can be expressed as:

$$\Delta d = x_1 \quad \text{(S16)}$$

By substituting Eq. (S16) into Eq. (S15), F_{dc} is transformed into:

$$F_{\mathrm{dc}} = \frac{4C_1V_{\mathrm{dc}}^2}{d^2}x_1 + \frac{8C_1V_{\mathrm{dc}}^2}{d^4}x_1^3 \quad \text{(S17)}$$

Here, the first term in Eq. (S17) is the linear term caused by the electrostatic force of, which only causes the negative stiffness k_1 of ω_1 in Eq. (S1) and does not contribute to the Duffing nonlinearity. However, the second term in Eq. (S17) forms the Duffing cubic term and contributes α_1. k_1 and α_1 can be expressed as:

$$\begin{aligned} k_1 &= \frac{4C_1V_{\mathrm{dc}}^2}{d^2} \\ \alpha_1 &= \frac{8C_1V_{\mathrm{dc}}^2}{d^4} \end{aligned} \quad \text{(S18)}$$

According to Eq. (S18), α_1 is not only affected by the mechanical design of the MEMS resonator, but also V_{dc} applied to the electrodes. Thus, the Duffing nonlinearity can be easily controlled by adjusting V_{dc}, which is useful for discussing the interaction mechanism between coherent energy transfer and Duffing nonlinear regime.

2.3 Model of dynamical coupling

The coherent energy transfer in our devices can be mathematically analyzed by coupling the mode of interest (ω_1), including its Duffing nonlinearity, with the phonon cavity mode (ω_2) [40]. In our study, the phonon cavity mode ω_2 is un-driven, and therefore can be described using a linear resonator. The two equations are coupled to each other through linear interactions [10]. Thus, the equations of motion are given by:

$$\begin{aligned} &\ddot{x}_1+\omega_1^2 x_1=\varepsilon\left[-2\gamma_1\dot{x}_1-2\left(c_{11}x_1+c_{12}x_2\right)\cos\left(\omega_{\mathrm{p}}t\right)+\alpha_1 x_1^3+F_{\mathrm{d}}\cos\left(\omega_{\mathrm{d}}t\right)\right] \\ &\ddot{x}_2+\omega_2^2 x_2=\varepsilon\left[-2\gamma_2\dot{x}_2-2\left(c_{21}x_1+c_{22}x_2\right)\cos\left(\omega_{\mathrm{p}}t\right)\right] \end{aligned} \tag{S19}$$

where x_{i} (i = 1, 2) is the displacement of the i-th oscillator, ω_{i} is the natural frequency, $\gamma_{\mathrm{i}} = 1/\tau_{\mathrm{i}}$ is the energy dissipation rate where τ_{i} is the decay time constant, c_{ij} (j = 1, 2) is the parametric (i = j) and coupling (i $\neq$ j) coefficient, respectively. $c_{12} = c_{21} = c$ is the linear coupling coefficient, which is obtained by matching the experiment results. α_1 is the Duffing cubic coefficient, which is mainly dominated by capacitive nonlinearity. ω_{p} and ω_{d} is the frequency of parametric excitation and harmonic driving, F_{d} is the amplitude of harmonic driving torque. Note that all these terms are normalized by the effective mass of the oscillator. The factor ε is formally introduced as a small and dimensionless parameter, which can be used to obtain the approximate solution.

2.4 Multi-scale approximation

2.4.1 Red parametric excitation

In order to obtain the stationary solution of Eq. (S19), multi-scale approximation is used. The solution of Eq. (S19) is expected to depend on time through two different scales. On the one hand, the fast oscillations have frequencies of the same order as ω_1, ω_2, ω_{p} and ω_{d}. On the other hand, the oscillation amplitudes and phases change more slowly. Thus, by using the lowest approximation order, the solution can be expressed in the form:

$$\begin{aligned} &x_1\left(T_0,T_1\right)=x_{10}\left(T_0,T_1\right)+\varepsilon x_{11}\left(T_0,T_1\right) \\ &x_2\left(T_0,T_1\right)=x_{20}\left(T_0,T_1\right)+\varepsilon x_{21}\left(T_0,T_1\right) \\ &x_{10}=\frac{1}{2}a_1\left(T_1\right)e^{i\left[\omega_1 T_0+\beta_1\left(T_1\right)\right]}+cc \\ &x_{20}=\frac{1}{2}a_2\left(T_1\right)e^{i\left[\omega_2 T_0+\beta_2\left(T_1\right)\right]}+cc \end{aligned} \tag{S20}$$

where x_{ij} (i = 1, 2; j = 0, 1) are the different parts of x_{i}; a_{i} and β_{i} are real, which depend on T_1 only, cc stands for the conjugate of the preceding terms. By substituting Eq. (S20) into Eq. (S19) and equating the coefficient of ε on both sides, we obtain:

$$\begin{aligned} &D_0^2 x_{11}+\omega_1^2 x_{11}=-2D_0D_1x_{10}-2\gamma_1 D_0 x_{10}-2\left(c_{11}x_{10}+c_{12}x_{20}\right)\cos\left(\omega_{\mathrm{p}}t\right)+\alpha_1 x_{10}^3+F_{\mathrm{d}}\cos\left(\omega_{\mathrm{d}}t\right) \\ &D_0^2 x_{21}+\omega_1^2 x_{21}=-2D_0D_1x_{20}-2\gamma_2 D_0 x_{20}-2\left(c_{21}x_{10}+c_{22}x_{20}\right)\cos\left(\omega_{\mathrm{p}}t\right) \end{aligned} \tag{S21}$$

When $\omega_{\mathrm{p}} \approx \omega_{\mathrm{red}}$, we introduce the detuning parameters σ_{p} and σ_{d} to express the parametric pump and harmonic excitation:

$$\begin{aligned} \omega_{\mathrm{p}} &= \omega_2 - \omega_1 + \varepsilon\sigma_{\mathrm{p}} \\ \omega_{\mathrm{d}} &= \omega_1 + \varepsilon\sigma_{\mathrm{d}} \end{aligned} \tag{S22}$$

By substituting Eq. (S22) into Eq. (S21) and eliminating secular terms from the particular solution of Eq. (S21), we obtain:

$$\begin{aligned} &-i\omega_1\left(a_1' + \gamma_1 a_1\right) + \omega_1 a_1 \beta_1' - \frac{1}{2} c_{12} a_2 e^{i\left(\beta_2 - \beta_1 - \sigma_{\mathrm{p}} T_1\right)} + \frac{3}{8}\alpha_1 a_1^3 + \frac{1}{2} F_d e^{i\left(\sigma_{\mathrm{d}} T_1 - \beta_1\right)} = 0 \\ &-i\omega_2\left(a_2' + \gamma_2 a_2\right) + \omega_2 a_2 \beta_2' - \frac{1}{2} c_{21} a_1 e^{i\left(\beta_1 - \beta_2 + \sigma_{\mathrm{p}} T_1\right)} = 0 \end{aligned} \tag{S23}$$

Then we separate the result into its real and imaginary parts and obtain:

$$\begin{aligned} &8\omega_1 a_1 \beta_1' + 3\alpha_1 a_1^3 - 4c_{12} a_2 \cos\left(\beta_2 - \beta_1 - \sigma_{\mathrm{p}} T_1\right) + 4F_{\mathrm{d}} \cos\left(\sigma_{\mathrm{d}} T_1 - \beta_1\right) = 0 \\ &2\omega_1 a_1' + 2\gamma_1 \omega_1 a_1 + c_{12} a_2 \sin\left(\beta_2 - \beta_1 - \sigma_{\mathrm{p}} T_1\right) - F_{\mathrm{d}} \sin\left(\sigma_{\mathrm{d}} T_1 - \beta_1\right) = 0 \\ &2\omega_2 a_2 \beta_2' - c_{21} a_1 \cos\left(\beta_2 - \beta_1 - \sigma_{\mathrm{p}} T_1\right) = 0 \\ &2\omega_2 a_2' + 2\gamma_2 \omega_2 a_2 - c_{21} a_1 \sin\left(\beta_2 - \beta_1 - \sigma_{\mathrm{p}} T_1\right) = 0 \end{aligned} \tag{S24}$$

Eq. (S24) can be transformed into an autonomous system (i.e., one in which T_1 does not appear explicitly) by letting:

$$\begin{aligned} \theta_1 &= \beta_2 - \beta_1 - \sigma_{\mathrm{p}} T_1 \\ \theta_2 &= \sigma_{\mathrm{d}} T_1 - \beta_1 \end{aligned} \tag{S25}$$

and steady-state motion takes place when:

$$a_{\mathrm{i}}' = \theta_{\mathrm{i}}' = 0 \tag{S26}$$

By substituting Eq. (S26) into Eq. (S24), we obtain:

$$\begin{aligned} &8\omega_1 a_1 \sigma_{\mathrm{d}} + 3\alpha_1 a_1^3 - 4c_{12} a_2 \cos\theta_1 + 4F_{\mathrm{d}} \cos\theta_2 = 0 \\ &2\gamma_1 \omega_1 a_1 + c_{12} a_2 \sin\theta_1 - F_{\mathrm{d}} \sin\theta_2 = 0 \\ &2\omega_2 a_2\left(\sigma_{\mathrm{p}} + \sigma_{\mathrm{d}}\right) - c_{21} a_1 \cos\theta_1 = 0 \\ &2\gamma_2 \omega_2 a_2 - c_{21} a_1 \sin\theta_1 = 0 \end{aligned} \tag{S27}$$

As the steady-state solution is approximated by the lowest order, the solution of mode 1 can be deduced according to formula 1 and formula 3 in Eq. (S20):

$$x_1 \approx x_{10} = a_1 \cos\left(\omega_1 t + \beta_1\right) \tag{S28}$$

Therefore, the amplitude response of mode 1 when $\omega_{\mathrm{p}} \approx \omega_{\mathrm{red}}$ can be numerically found by using a standard multidimensional Newton-Raphson algorithm [41] to solve Eq. (S27).

Also, from the third and fourth of Eq. (S27) it can be seen that the amplitude of mode 2 can be expressed as:

$$a_2 = \frac{c a_1}{2\omega_2 \sqrt{\gamma_2^2 + \left(\sigma_{\mathrm{p}} + \sigma_{\mathrm{d}}\right)^2}} \tag{S29}$$

It can be seen form Eq. (S29) that the maximum value of a_2 can be obtained when $\sigma_{\mathrm{p}} + \sigma_{\mathrm{d}} = 0$, or equivalently $\omega_{\mathrm{d}} + \omega_{\mathrm{p}} = \omega_2$, as expected for pumping in the cavity red sideband.

2.4.2 Blue parametric excitation

When $\omega_{\mathrm{p}} \approx \omega_{\mathrm{blue}}$, the solution process is similar. Here, the detuning parameters changes to:

$$\begin{aligned} \omega_{\mathrm{p}} &= \omega_2 + \omega_1 + \varepsilon\sigma_{\mathrm{p}} \\ \omega_{\mathrm{d}} &= \omega_1 + \varepsilon\sigma_{\mathrm{d}} \end{aligned} \tag{S30}$$

By substituting Eq. (S30) into Eq. (S21) and eliminating secular terms from the particular solution of Eq. (S21), we obtain:

$$\begin{aligned} &-i\omega_1\left(a_1' + \gamma_1 a_1\right) + \omega_1 a_1 \beta_1' - \frac{1}{2} c_{12} a_2 e^{i\left(\sigma_{\mathrm{p}} T_1 - \beta_1 - \beta_2\right)} + \frac{3}{8}\alpha_1 a_1^3 + \frac{1}{2} F_{\mathrm{d}} e^{i\left(\sigma_{\mathrm{d}} T_1 - \beta_1\right)} = 0 \\ &-i\omega_2\left(a_2' + \gamma_2 a_2\right) + \omega_2 a_2 \beta_2' - \frac{1}{2} c_{21} a_1 e^{i\left(\sigma_{\mathrm{p}} T_1 - \beta_1 - \beta_2\right)} = 0 \end{aligned} \tag{S31}$$

Then we separate the result into its real and imaginary parts and obtain:

$$\begin{aligned} &8\omega_1 a_1 \beta_1' + 3\alpha_1 a_1^3 - 4c_{12} a_2 \cos\left(\sigma_{\mathrm{p}} T_1 - \beta_1 - \beta_2\right) + 4F_{\mathrm{d}} \cos\left(\sigma_{\mathrm{d}} T_1 - \beta_1\right) = 0 \\ &2\omega_1 a_1' + 2\gamma_1 \omega_1 a_1 + c_{12} a_2 \sin\left(\sigma_{\mathrm{p}} T_1 - \beta_1 - \beta_2\right) - F_{\mathrm{d}} \sin\left(\sigma_{\mathrm{d}} T_1 - \beta_1\right) = 0 \\ &2\omega_2 a_2 \beta_2' - c_{21} a_1 \cos\left(\sigma_{\mathrm{p}} T_1 - \beta_1 - \beta_2\right) = 0 \\ &2\omega_2 a_2' + 2\gamma_2 \omega_2 a_2 + c_{21} a_1 \sin\left(\sigma_{\mathrm{p}} T_1 - \beta_1 - \beta_2\right) = 0 \end{aligned} \tag{S32}$$

Eq. (S32) can be transformed into an autonomous system by letting:

$$\begin{aligned} \theta_1 &= \sigma_{\mathrm{p}} T_1 - \beta_1 - \beta_2 \\ \theta_2 &= \sigma_{\mathrm{d}} T_1 - \beta_1 \end{aligned} \tag{S33}$$

and steady-state motion takes place when:

$$a_{\mathrm{i}}' = \theta_{\mathrm{i}}' = 0 \tag{S34}$$

By substituting Eq. (S34) into Eq. (S32), we obtain:

$$\begin{aligned} &8\omega_1 a_1 \sigma_{\mathrm{d}} + 3\alpha_1 a_1^3 - 4c_{12} a_2 \cos\theta_1 + 4F_{\mathrm{d}} \cos\theta_2 = 0 \\ &2\gamma_1 \omega_1 a_1 + c_{12} a_2 \sin\theta_1 - F_{\mathrm{d}} \sin\theta_2 = 0 \\ &2\omega_2 a_2 \left(\sigma_{\mathrm{p}} - \sigma_{\mathrm{d}}\right) - c_{21} a_1 \cos\theta_1 = 0 \\ &2\gamma_2 \omega_2 a_2 + c_{21} a_1 \sin\theta_1 = 0 \end{aligned} \tag{S35}$$

Thus, the amplitude response of mode 1 when $\omega_p \approx \omega_{blue}$ can be numerically found from Eq. (S35).

Also, from the third and fourth of Eq. (S35) it can be seen that the amplitude of mode 2 can be expressed as:

$$a_2 = \frac{ca_1}{2\omega_2\sqrt{\gamma_2^2 + \left(\sigma_p - \sigma_d\right)^2}} \tag{S36}$$

It can be seen form Eq. (S36) that the maximum value of a_2 can be obtained when $\sigma_p - \sigma_d = 0$, or equivalently $\omega_d + \omega_2 = \omega_p$, as expected for pumping in the cavity blue sideband.

2.4.3 Summary of numerical parameters

The parameters used in the model for calculating the stationary solutions are listed below in Table S1.

TABLE S1. Parameters used for calculating the stationary solutions.

Coefficient	Value	unit
ω_1	$2\pi \times 6869.1$	Hz
ω_2	$2\pi \times 16649.6$	Hz
γ_1	1.78	s^{-1}
γ_2	4.18	s^{-1}
C_1	1.53	pF
d	2	μm
V_{dc}	2.5	V
α_1	4.32×10^{22}	$N.m^{-3}$
c	$10^8 \sim 10^9$	$N.m^{-1}$

3. ADDITIONAL EXPERIMENT AND SIMULATION RESULTS

3.1 Evolution of the phonon cavity from linear to nonlinear regime

The evolution of the resonant behavior in our phonon cavity, as depth of nonlinearity is gradually increased, in carefully studied both experimentally and numerically. Using the numbers for the red pump case as an example (blue pump is similar): in Fig. S5 we show the results for intermediate driving (V_d = 0.5 mV$_{pk}$), as the phonon cavity system transitions from linear operation (Figs. 2a-b and 3a in the Main Text, V_d = 0.05 mV$_{pk}$) to deep nonlinear operation (Figs. 2i-j and 3e in the Main Text, V_d = 1.5 mV$_{pk}$). It can be seen that with intermediate driving amplitude, the phonon cavity system starts to develop nonlinear responses, and the features gradually grow (such as the spike-like feature in the blue-pump case), eventually evolving into the deep-nonlinear case.

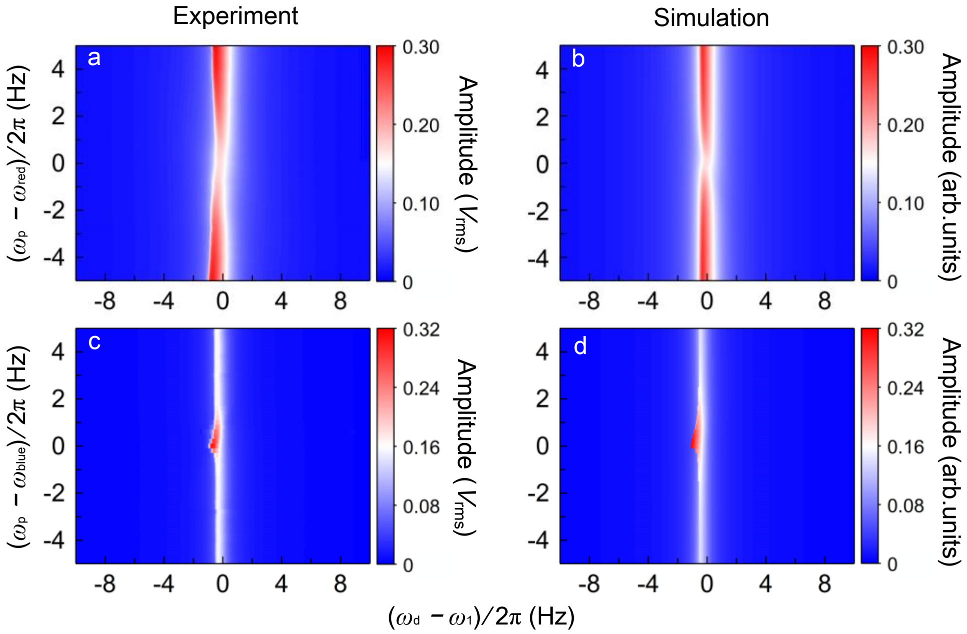

FIG. S5. The resonant response of the phonon cavity system under intermediate driving. Data shown are from downward frequency sweeps. (a) The experiment and (b) simulation results when pumping near the red sideband. The experiment and simulation parameters are the same: driving voltage V_d = 0.5 mV$_{pk}$, pump voltage V_p = 5.0 V$_{pk}$, and are plotted using $\omega_1 = 2\pi \times 6886.0$ Hz, $\omega_{red} = 2\pi \times 9592.0$ Hz. (c) The experiment and (d) simulation results when pumping near the blue sideband. The experiment and simulation parameters are the same: driving voltage V_d = 0.2 mV$_{pk}$, the pump voltage V_p = 2.0 V$_{pk}$. and are plotted using $\omega_1 = 2\pi \times 6958.0$ Hz, $\omega_{blue} = 2\pi \times 23581.2$ Hz. The frequency steps of all plots are $\Delta\omega_d = 2\pi \times 0.05$ Hz, and $\Delta\omega_p = 2\pi \times 0.2$ Hz.

3.2 Frequency digitization and amplification when $\omega_p \approx \omega_{red}$

Similar to the case of blue sideband (Fig. 4 in the Main Text), frequency digitization and amplification can also be realized when pumping new the red sideband (Fig. S6). One distinct feature in the red sideband case is that the dip-like feature replaces the spike-like feature in the blue-pumped case, and therefore the signs of frequency shift in ω_b is opposite. The effects can also be modulated by pump strength and depth of nonlinearity, as shown in Fig. S6.

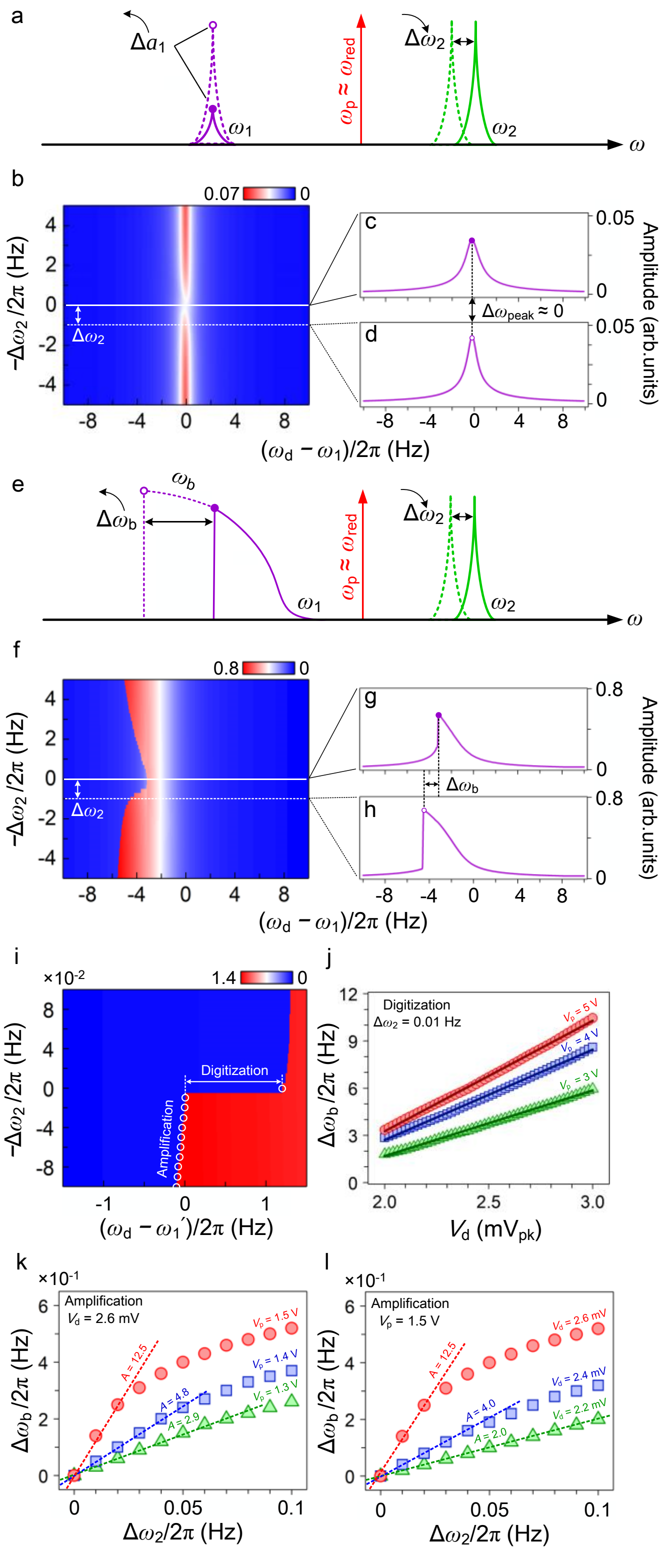

FIG. S6 Signal transduction and amplification in the 2-DOF phonon cavity system, when pumping near the red sideband. (a) Schematic illustration of the phonon-cavity's response (purple signal) to a shift in the input signal (green) under linear vibration, when the pump signal (red) is aligned with the cavity's red sideband. (b) 2D colour plot of the phonon-cavity's response to the input perturbation under linear vibration. (c,d) Line plots taken from different operation points in (b), showing negligible shift in resonance peak frequency ω_{peak}. (e) Schematic illustration of the phonon-cavity's response (purple signal) to a shift in the input signal (green) under nonlinear vibration, when the pump signal (red) is aligned with the cavity's red sideband. (f) 2D colour plot of the

phonon-cavity's response to the input perturbation under nonlinear vibration. (g,h) Line plots taken from different operation points in (f), showing significant shift in ω_b. (i) Illustrating the regions used for the two frequency-domain operations. (j) The output shift in ω_b for a given input shift $\Delta\omega_2$ = 0.01 Hz as functions of V_d, under different V_p values. (k) The output shift in ω_b versus input shift in ω_2 for different V_p values. Larger V_p corresponds to stronger parametric coupling. (l) The output shift in ω_b versus input shift in ω_2 for different V_d values. Larger V_d corresponds to deeper nonlinearity.

The numerical settings for producing the data in Fig. S6 are as follows:

In the linear operation (b), V_d = 0.05 mV_{pk}, V_p = 5.0 V_{pk}, $\omega_1 = 2\pi \times 6886.0$ Hz, and $\omega_p = 2\pi \times 9592.0$ Hz. The frequency steps are $\Delta\omega_d = 2\pi \times 0.05$ Hz, and $\Delta\omega_2 = 2\pi \times 0.2$ Hz. The line plots (c, d) are taken from data in (b) with $\Delta\omega_2 = 0$ (c) and $\Delta\omega_2 = 2\pi \times 1.0$ Hz (d).

In the nonlinear operation with downward sweep (f), V_d = 1.5 mV_{pk}, V_p = 5.0 V_{pk}, $\omega_1 = 2\pi \times 6886.0$ Hz, and $\omega_p = 2\pi \times 9592.5$ Hz. The frequency steps are $\Delta\omega_d = 2\pi \times 0.05$ Hz, and $\Delta\omega_2 = 2\pi \times 0.2$ Hz. The line plots (g, h) taken from data in (d) when $\Delta\omega_2 = 0$ (g) and $\Delta\omega_2 = 2\pi \times 1.0$ Hz (h).

In the zoom-in plot (i), V_d = 2.6 mV_{pk}, V_p = 1.8 V_{pk}, $\omega_1 = 2\pi \times 6874.50$ Hz, and $\omega_p = 2\pi \times 9602.57$ Hz. The frequency steps are $\Delta\omega_d = 2\pi \times 0.01$ Hz, $\Delta\omega_2 = 2\pi \times 0.01$ Hz.

The data of digitization in (j) are calculated for V_p = 3.0, 4.0, 5.0 V_{pk} when V_d is scanned from 2.0 mV_{pk} to 3.0 mV_{pk}. The step of V_d is 0.1 mV_{pk}.

The data of amplification in (k) are calculated for V_d is 2.6 mV_{pk} and V_p = 1.3, 1.4, 1.5 V_{pk}. The step of $\Delta\omega_2$ is $2\pi \times 0.01$ Hz.

The data of amplification in (l) are calculated for V_p is 1.5 V_{pk} and V_d = 2.2, 2.4, 2.6 mV_{pk}. The step of $\Delta\omega_2$ is $2\pi \times 0.01$ Hz.

3.3 Evolution of the frequency digitization and amplification functions

The frequency digitization and amplification can be continuously modulated by depth of nonlinearity and pump depth (Figs. 4 j-l in the Main Text, and Figs. S6 j-l). Here we take a closer look in the 2D colour plots for such evolution as function of pump strength, by zooming into the parameter space of interest (corresponding to the plotting areas of Fig. 4i in the Main Text and Fig. S6i). In comparison, the evolution of the overall resonant behavior with the depth of nonlinearity is presented in Fig. S5 (corresponding to the plotting areas of Figs. 4b, 4f in the Main Text and Figs. S6b, S6f).

We show in Fig. S7 such evolution for both the red-pumped (a-c) and blue-pumped (d-f) cases. It can be seen that stronger pump strength corresponds to sharper dip-like and spike-like features, and the sharpness of such features, corresponding the magnitude of the frequency digitization and gain of the amplification, can be continuously tuned by the pump strength.

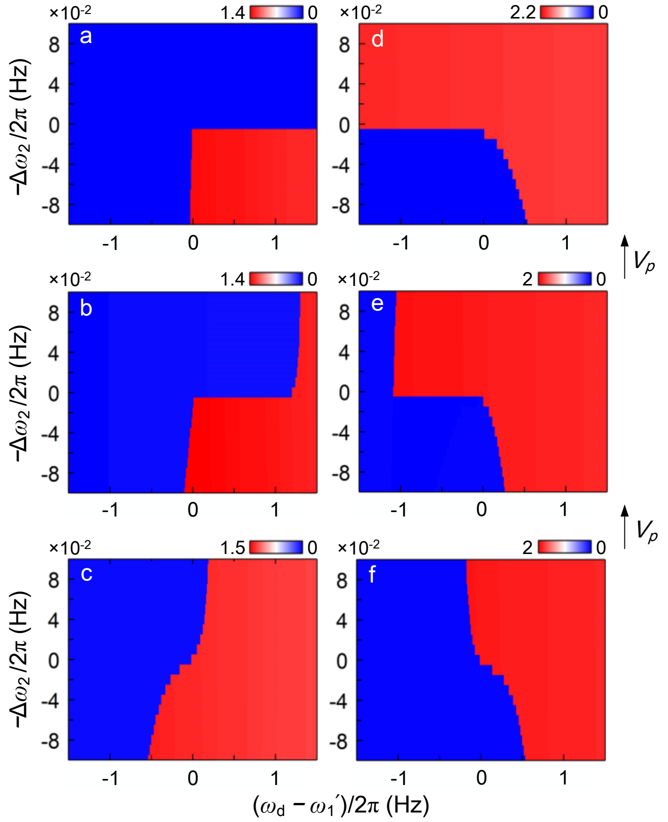

FIG. S7. The evolution process of frequency digitization and amplification with pump strength. (a-c) The results when pumping near the red sideband. (a) V_d = 2.6 mV$_{pk}$, V_p = 5.0 V$_{pk}$, $\omega_1 = 2\pi \times 6874.65$ Hz, and $\omega_p = 2\pi \times 9595.90$ Hz. (b) V_d = 2.6 mV$_{pk}$, V_p = 1.8 V$_{pk}$, $\omega_1 = 2\pi \times 6874.01$ Hz, and $\omega_p = 2\pi \times 9602.57$ Hz. (c) V_d = 2.6 mV$_{pk}$, V_p = 1.5 V$_{pk}$, $\omega_1 = 2\pi \times 6874.46$ Hz, and $\omega_p = 2\pi \times 9603.17$ Hz. (d-f) The results when pumping near the blue sideband. (d) V_d = 3.0 mV$_{pk}$, V_p = 2.0 V$_{pk}$, $\omega_1 = 2\pi \times 6926.74$ Hz, and $\omega_p = 2\pi \times 23546.27$ Hz. (e) V_d = 3.0 mV$_{pk}$, V_p = 0.6 V$_{pk}$, $\omega_1 = 2\pi \times 6929.52$ Hz, and $\omega_p = 2\pi \times 23552.51$ Hz. (f) V_d = 3.0 mV$_{pk}$, V_p = 0.5 V$_{pk}$, $\omega_1 = 2\pi \times 6930.33$ Hz, and $\omega_p = 2\pi \times 23553.08$ Hz. In all measurements, the frequency scanning steps are the same: $\Delta\omega_d = 2\pi \times 0.01$ Hz, $\Delta\omega_2 = 2\pi \times 0.01$ Hz. Note that the plot center frequencies are slightly adjusted for each panel for easy comparison across the different measurement conditions.

3.4 Amplification and digitization operations with fast readout using PLL

When carrying out the PLL measurements, the reference phase of mode 1 is locked at a carefully chosen value. This is because the sudden, discontinuous downward jump at ω_b can cause failure of the phase lock loop. Therefore, the reference phase is chosen to be less than 90 °in order for the operating point to stay away from the downward jump, and the operating frequency is thus slightly blue shifted from the bifurcation point ω_b. In actual experiments, the exact value of reference phase is carefully adjusted for each different set of experimental parameters of V_d and V_p to ensure proper operation of the PLL, and the value ranges between 72.5 °and 81.9 °for all PLL experiments. Specifically, for the measurement shown in Fig. 5 (b) the reference phase is 72.5 °, and for the measurement shown in Fig. S9 (b) the reference phase is 79.1 °.

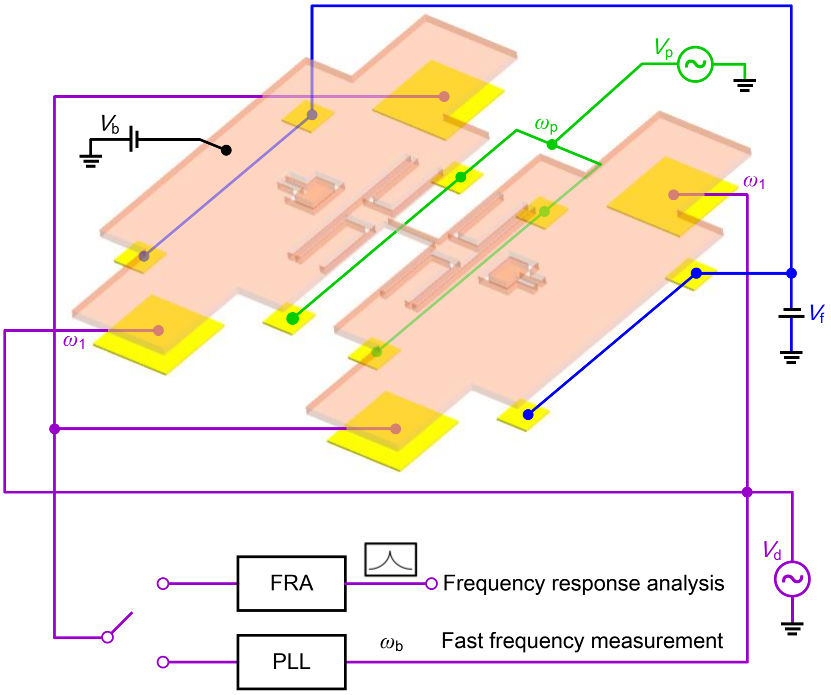

FIG. S8. Schematic diagram of the experiment setup for measuring the frequency-shift-based sensing functions.

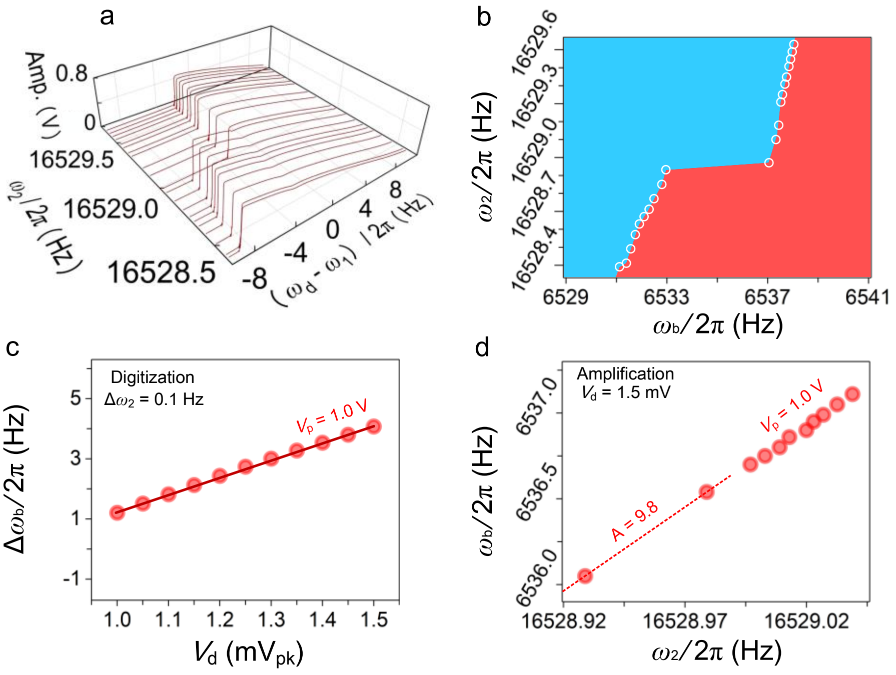

FIG. S9. Experimental demonstration of signal transduction and amplification using the nonlinear phonon-cavity system at red pump. (a) The frequency response curves as functions of ω_2 in downward frequency sweep measurements. (b) Fast measurement of ω_b as functions of ω_2. The white circles are the PLL measured data, and the solid colors on both sides offer visual cues to the eye. (c) The output shift in ω_b as functions of ω_2 in the digitization region measured by PLL. (d) The output ω_b as functions of ω_2 in the amplification region measured by PLL.

The measurement settings for producing Fig. S9 are as follows:

In the nonlinear operation at red pump with downward sweep (a), V_d = 1.5 mV$_{pk}$, V_p = 1.0 V$_{pk}$, $\omega_1 = 2\pi \times 6540.0$ Hz, and $\omega_p = 2\pi \times 9982.1$ Hz. The steps are $\Delta\omega_d = 2\pi \times 0.02$ Hz, and ΔV_f = 0.05V$_{pk}$.

When using PLL to test the phonon-cavity system at red pump (b), V_d = 1.5 mV$_{pk}$, V_p = 1.0 V$_{pk}$, and $\omega_p = 2\pi \times 9982.1$ Hz. V_f is scanned from 0 to 1V$_{pk}$ and the step of V_f is 0.05V$_{pk}$.

The experiment of digitization at red pump in (c), V_p = 1.0 V$_{pk}$ when V_d is scanned from 1.0 mV$_{pk}$ to 1.5 mV$_{pk}$. The step of V_d is 0.05 mV$_{pk}$.

The experiment of amplification at red pump in (d), V_d = 1.5 mV$_{pk}$, V_p = 1.0 V$_{pk}$, and $\omega_p = 2\pi \times$ 9982.1 Hz. V_f is scanned from 0.445 to 0.495 V$_{pk}$ and the step of V_f is 5 mV$_{pk}$.

3.5 Frequency noise of the phonon-cavity system

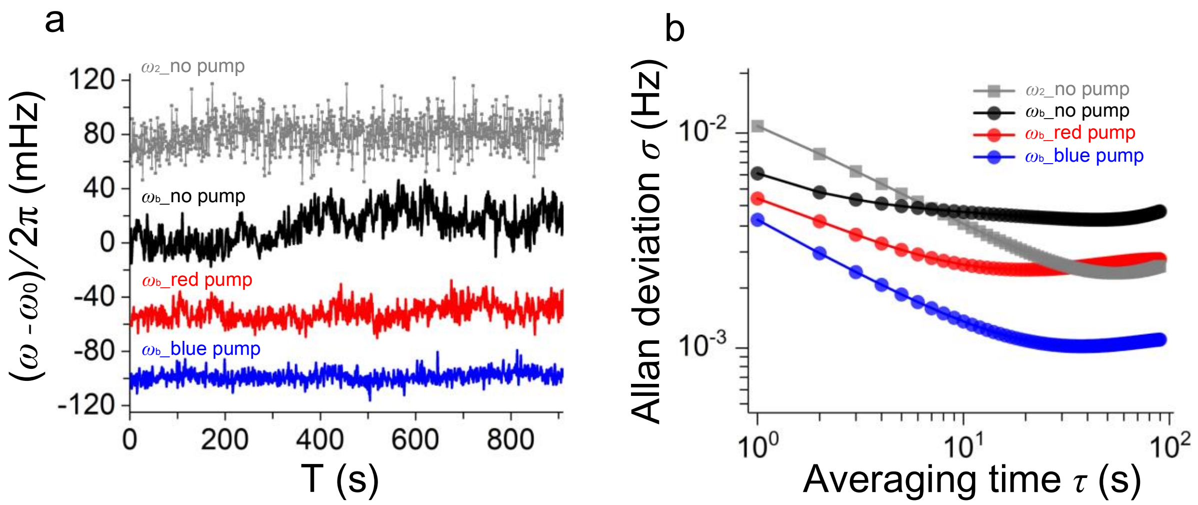

FIG. S10. Measurement results of the frequency noise. (a) The time domain fluctuation of ω_2 and ω_b: ω_2 with no pump (gray line with dots), ω_b with no pump (black line), ω_b with red pump (red line), and ω_b with blue pump (blue line). Note that the data are vertically offset for clarity. When measuring the frequency noise, V_d = 1.0 mV$_{pk}$, V_p = 1.5 V$_{pk}$, $\omega_{blue} = 2\pi \times 23.0558$ Hz, and $\omega_{red} = 2\pi \times 9971.1$ Hz. The reference frequency ω_0 values for the four different data series are: 16530.27 Hz (no pump of ω_2), 6546.72 Hz (no pump of ω_b), 6537.00 Hz (blue pump of ω_b), and 6540.13 Hz (red pump of ω_b), respectively. The sampling rate of the frequency noise measurement is 1 Hz and the each test lasts 900 s. (b) Allan deviation of of ω_2 and ω_b: ω_2 with no pump (gray line and squares), ω_b with no pump (black line and circles), ω_b with red pump (red line and circles), and ω_b with blue pump (blue line and circles).

TABLE S2. Results of frequency instability and random walk.

Condition	Random walk (Hz/√Hz)	Instability (Hz)
ω_2 no pump	1.18×10^{-2}	9.09×10^{-4}
ω_b no pump	4.3×10^{-3}	6.3×10^{-3}
ω_b red pump	5.3×10^{-3}	2.3×10^{-3}
ω_b blue pump	3.8×10^{-3}	6.89×10^{-4}
